# Tocilizumab overcomes chemotherapy resistance in mesenchymal stem-like breast cancer by negating autocrine IL-1A induction of IL-6

**DOI:** 10.1038/s41523-021-00371-0

**Published:** 2022-03-08

**Authors:** Andrew W. Chung, Anthony J. Kozielski, Wei Qian, Jianying Zhou, Ann C. Anselme, Alfred A. Chan, Ping-Ying Pan, Delphine J. Lee, Jenny C. Chang

**Affiliations:** 1 Texas A&M University Health Science Center, Bryan, TX 77807 USA; 2 Houston Methodist Research Institute, Houston, TX 77030 USA; 3 Houston Methodist Cancer Center, Houston, TX 77030 USA; 4 The Lundquist Institute, Torrance, CA 90502 USA; 5 David Geffen School of Medicine at UCLA, Los Angeles, CA 90095 USA

**Keywords:** Breast cancer, Cell biology

## Abstract

Triple-negative breast cancer (TNBC) patients with mesenchymal stem-like (MSL) subtype have responded poorly to chemotherapy whereas patients with basal-like 1 (BL1) subtype achieved the best clinical response. In order to gain insight into pathways that may contribute to the divergent sensitivity to chemotherapy, we compared the inflammatory profile of the two TNBC subtypes treated with docetaxel. Cellular signaling analysis determined that docetaxel activated MAPK pathway in MSL TNBCs but not BL1 TNBCs. The subsequent MAPK pathway activation in MSL TNBCs led to an IL-1A mediated cascade of autocrine inflammatory mediators including IL-6. Utilizing the humanized IL-6R antibody, tocilizumab, our in vitro and in vivo data show that MSL TNBCs treated with tocilizumab together with chemotherapy results in delayed tumor progression compared to MSL TNBCs treated with docetaxel alone. Our study highlights a molecular subset of TNBC that may be responsive to tocilizumab therapy for potential translational impact.

## Introduction

Triple-negative breast cancers (TNBC) account for 15–20% of all breast cancer cases and ~1 million new TNBC cases are diagnosed globally^1,2^. Unfortunately, TNBC patients do not benefit from endocrine and growth factor receptor neutralizing therapies as they are receptor-independent cancer cells. TNBC remains very difficult to treat and most patients develop recurrence or distant metastasis^3,4^. Indeed, metastatic TNBC patients have a grim overall survival of 9–12 months undergoing conventional therapies^5,6^. Furthermore, the heterogeneity of TNBC results in numerous chemoresistance pathways that are not yet fully delineated. This necessitates ongoing research to elucidate the cellular biology of TNBC to develop novel therapeutics to improve patient survival.

Previous molecular signaling pathway investigations by others identified that TNBC can be subdivided into distinct subtypes based on gene expression signatures^7^. The basal-like 1 (BL1) subtype had genes enriched in cell cycle and division^7^, and TNBC patients with BL1 tumors achieved the best clinical responses^8,9^. In contrast, the mesenchymal stem-like (MSL) subtype had gene enrichment in hallmark cancer pathways: metastasis, angiogenesis, and stem cell^7^. Patients with MSL subtype are more chemoresistant and achieve one of the worst clinical response rates^8,9^. Although this TNBC subtyping method is reproducible and early studies show that BL1 tumors are susceptible to antineoplastic agents, it is not yet clear what resistance mechanisms are utilized by MSL tumors in response to chemotherapy. Therefore, elucidating MSL chemoresistance mechanisms will allow integration of targeted therapies aimed to neutralize unique MSL pathways to complement conventional therapy.

Numerous inflammatory cytokines have been shown to activate pro-tumor pathways in various cancers^10–14^. It has also been established that various chemotherapy regimens correlate with elevated cytokine levels in cancer patients^15–17^. Therefore, it is plausible that the resulting chemotherapy-associated inflammatory cytokines may promote resistance mechanisms in TNBC cells. Given that MSL TNBC had gene enrichment in the inflammatory NF-kB pathway^7^, we hypothesized that MSL TNBCs may resist docetaxel therapy with self-induced proinflammatory cytokines.

As single agent taxanes are frontline therapy for TNBC patients^18^, we investigated potential chemoresistant mechanisms by analyzing the inflammatory profile differences between MSL and BL1 TNBCs treated with docetaxel. Our results suggest that docetaxel-treated MSL TNBCs initiate an autocrine IL-1A circuit to promote tumor production of IL-6. This mechanism is unique to MSL TNBCs and not observed in BL1 TNBCs. IL-6 is a well-established inducer of STAT3 signaling^19,20^, and STAT3 signaling mediates cancer proliferation^21–23^. Therefore, targeting tumor-derived IL-6 with pharmacological inhibitors may negate chemoresistance pathways in docetaxel-treated MSL TNBCs. We tested our hypothesis by investigating the therapeutic efficacy of tocilizumab, a humanized anti-IL6R antibody with FDA approval for various auto-immune diseases^24,25^. This study aims to elucidate the upstream pathway promoting autocrine cytokine production in MSL TNBCs, and investigate the potential benefit of a novel regimen combining tocilizumab with docetaxel against different TNBC subtypes.

## Results

### Differential inflammatory gene expression in MSL vs. BL1 TNBC cell lines

**Fig. 1:**
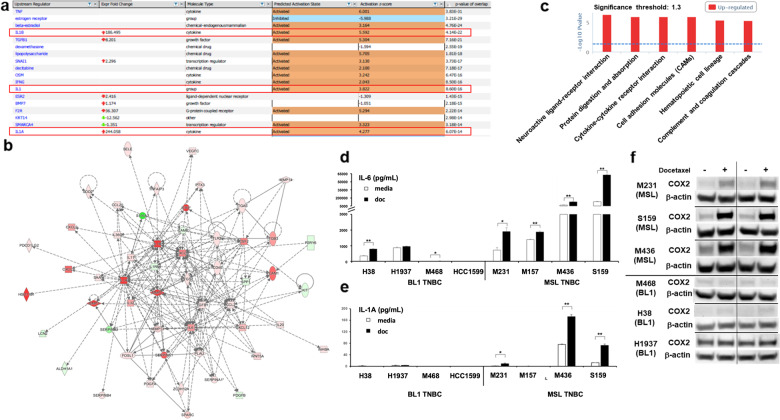
MSL TNBCs have predicted IL-1A mediated signaling, which is absent in BL1 TNBCs. Four MSL TNBC and four BL1 TNBC cell lines were treated in presence or absence of docetaxel (4 ng/ml) for 48 h and RNA sequencing was performed. **a** IPA upstream regulator analysis of fold change ratio (cutoff value of 5 or −5) comparing averaged RPKM from four docetaxel-treated MSL TNBC to averaged RPKM from four docetaxel-treated BL1 TNBC. **b** IPA pathway builder of IL1A upstream regulator network overlayed with dataset from **a** (fold change ratio comparing averaged MSL docetaxel treated to averaged BL1 docetaxel-treated samples). **c** InnateDB analysis of aggregate 16 samples (four MSL media, four BL1 media, four MSL doc and four BL1 doc) with a permutation test comparing MSL to BL1 (*p*-value < 0.05 and log2FC > 4). Shown are pathways upregulated in MSL TNBCs compared to BL1 TNBCs. **d** IL-6 and **e** IL-1A average ± SEM ELISA results from supernatants collected 48 h from each indicated human TNBC cell lines treated in presence or absence of docetaxel at 4 ng/ml. Each cell line was tested with three biological replicates and results are representative of at least two independent experiments. **f** Each cell line was treated in the presence or absence of docetaxel (4 ng/ml), and whole-cell lysate was collected after 48 h and western blot for COX-2 and b-actin was performed for all indicated samples. Results are representative of at least two independent experiments. Statistical analysis performed with unpaired *t*-test: *p < 0.05; **p < 0.01.

RNA sequencing was performed on four human MSL TNBC cell lines and four human BL1 TNBC cell lines that were untreated or treated with docetaxel. Gene expression profile differences were compared between untreated MSL vs. BL1 TNBCs, and docetaxel-treated MSL vs. BL1 TNBCs. Ingenuity Pathway Analysis (IPA) demonstrated that MSL TNBCs had a higher inflammatory gene signature compared to BL1 TNBCs^7^. IPA predicted that the IL-1 cytokine family was upstream regulators in MSL TNBCs at baseline levels (Supplementary Fig. 1A) and after docetaxel (Fig. 1a), which was missing in the BL1 TNBC cell lines. IL-1A and IL-1B are both potent inflammatory cytokines with multi-faceted roles in homeostasis, immunity, and inflammatory diseases^26,27^. Pathogen-mediated activation is required for mature IL-1B and given that our model is in the context of “sterile” inflammation with the absence of microbial antigens, we hypothesized that upregulation of the IL-1B transcript would not result in biologically active IL-1B. We confirmed that all TNBC cell lines did not produce any detectable levels of mature IL-1B protein (data not shown). Next, we investigated the inflammatory network regulated by IL-1A in MSL TNBCs. IPA network pathway results showed that MSL TNBC gene expression profiles had an IL-1A regulated interaction with other inflammatory mediators such as IL-6, IL-8, CSF2, CXCL2, CXCL3, IL-32, IL-11 at both baseline levels (Supplementary Fig. 1B) and after docetaxel treatment (Fig. 1b). Given that we observed an upregulated cytokine network in MSL TNBCs, analysis was performed on a database for innate immune signaling pathways (InnateDB). Results indicated that MSL TNBCs had a significant upregulation of cytokine-cytokine receptor interaction compared to BL1 TNBCs (*p* < 0.05, Fig. 1c). In addition, heatmap analysis of the RNA sequencing results confirmed that MSL TNBCs had higher gene expression of various inflammatory mediators compared to their BL1 counterparts (Supplementary Fig. 1C), which were validated by confirmatory RT-PCR of select target genes (Supplementary Fig. 1D). Cumulatively, these data suggest that MSL TNBCs had an inflammatory gene expression profile that may be regulated by IL-1A in a mechanism that is absent in BL1 TNBCs.

### Docetaxel induces inflammatory mediators in MSL TNBCs and not BL1 TNBCs

Next, we confirmed our RNA sequencing results by measuring select inflammatory mediators following docetaxel treatment in vitro by examining protein induction. Docetaxel treatment induced statistically significant levels of IL-6 in MSL TNBCs ranging from ~2 to 63 ng/ml (Fig. 1d), whereas docetaxel induced IL-6 inconsistently from BL1 TNBCs ranging from not detectable to 1 ng/ml. Similarly, docetaxel treatment mediated statistically significant production of mature IL-1A in three out of four MSL TNBC cell lines (Fig. 1e) compared to a complete absence of detectable extracellular IL1A in BL1 TNBCs. COX-2 was also upregulated following docetaxel treatment in MSL TNBCs (Fig. 1f), which did not occur in BL1 TNBCs. As both mature and full-length forms of IL-1A are biologically active^26,27^, we determined the expression of pro IL-1A by western blot in the representative TNBC cell lines. In alignment with the extracellular IL-1A data (Fig. 1e), docetaxel induced pro IL-1A only in the surveyed MSL TNBCs compared to virtual absence in BL1 TNBCs (Supplementary Fig. 2). Because IL-6 is a well-characterized cytokine that is utilized for the benefit of various cancers^21–23^, we explored the biological relevance of this cytokine in TNBCs. The signaling component of IL-6 requires both the IL-6R and gp130 as the downstream signal transducer for subsequent Jak/STAT activation^28^. Although we found that all TNBC cell lines had ubiquitous expression of IL-6R (Supplementary Fig. 2), we also found that MSL TNBCs had a trend of higher expression of the gp130 signal transducer in comparison to BL1 TNBCs. Docetaxel however did not alter the expression of the IL-6 signaling component in any TNBC cell line. These data confirms that docetaxel augments an inflammatory cytokine profile unique to MSL TNBCs which is notably absent in BL1 TNBCs.

### MAPK pathway mediated IL-1A promotes the inflammatory cascade in MSL TNBCs

**Fig. 2:**
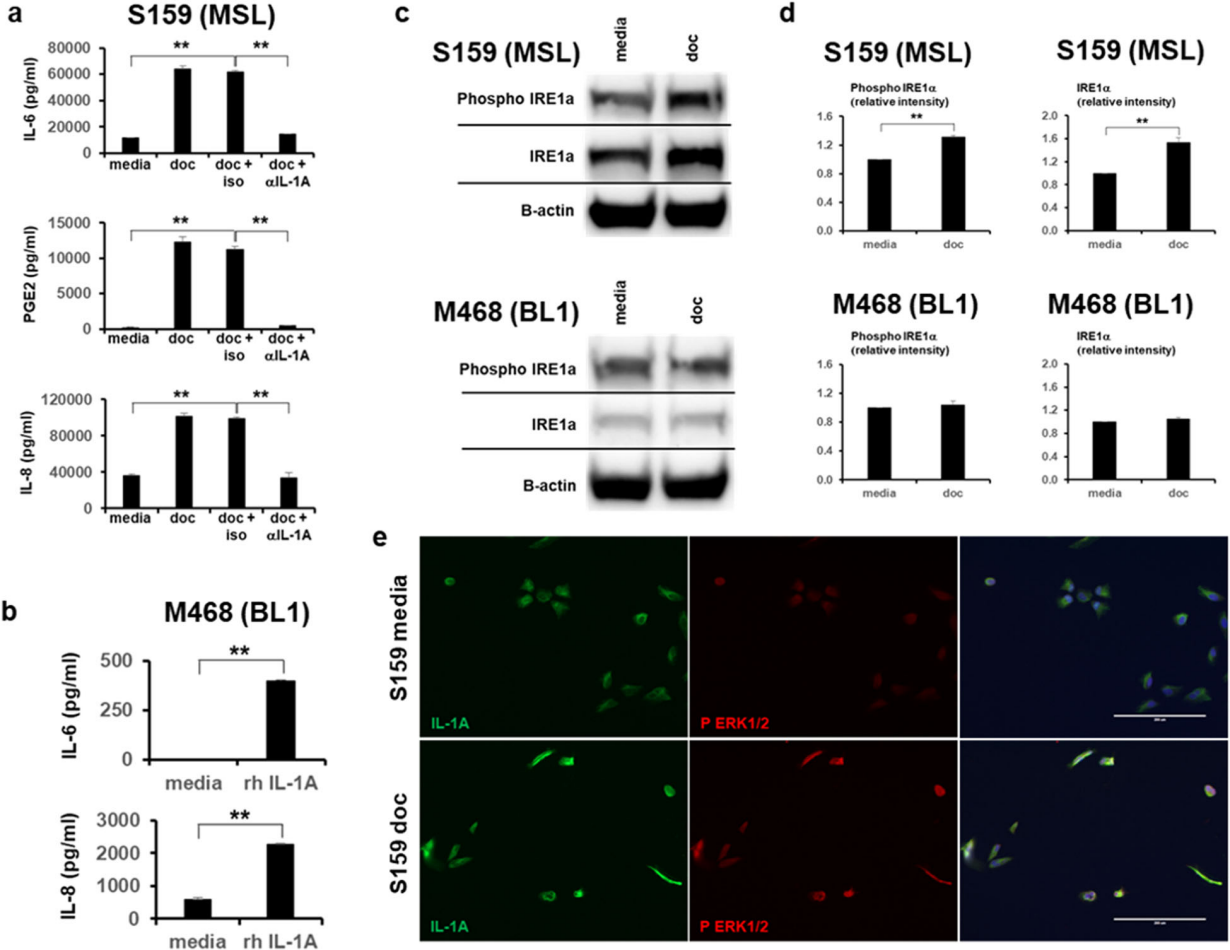
IL-1A is the upstream cytokine that promotes docetaxel mediated inflammation. **a** IL-6, PGE2, and IL-8 ELISA results from supernatants collected from human TNBC cell line treated 48 h in presence or absence of docetaxel (doc) at 4 ng/ml, NA/LE IL-1A mAb (1 μg/ml) or NA/LE isotype mIgG2a (1 μg/ml). **b** BL1 TNBC treated in presence or absence of recombinant human (rh) IL-1A (2 ng/ml) for 48 h and supernatants analyzed for IL-6 and IL-8 ELISAs. **a**, **b** Show average ± SEM from *n* = 3 biological replicates. **c** Indicated TNBCs were treated in the presence or absence of doc (4 ng/ml) for 48 h. WB from whole-cell lysates was performed, shown are representative images. **d** Average ± SEM densitometry analysis is from *n* = 3. **e** Shown are representative 20 X images (*n* = 3 biological replicates) from S159 (MSL) cells cultured 48 h in chambered slides with or without doc (4 ng/ml). Immunofluorescence staining for IL-1A (Alexa Fluor 488) and Phospho ERK1/2 (Alexa 546) was performed. Statistical analysis performed with unpaired *t*-test: *p < 0.05; **p < 0.01. All indicated experiments are representative of at least two independent experiments.

As our previous results indicated that IL-1A is an upstream in MSL TNBCs (Fig. 1a), we investigated whether elimination of IL-1A would diminish the inflammatory profile in MSL TNBCs. Two representative MSL TNBC cell lines, SUM-159 and MDA-MB-436, were treated with docetaxel in the presence or absence of IL-1A neutralizing antibody. IL-1A neutralizing antibody resulted in 72-77% reduction of docetaxel mediated production of IL-6 in the MSL TNBC cell lines versus isotype control (*p* < 0.001 in both cell lines, Fig. 2a and Supplementary Fig. 3A). IL-1A neutralizing antibody also resulted in virtual elimination of PGE2, the main metabolite of COX-2^29^, following docetaxel treatment in the MSL TNBC cell lines (*p* < 0.05 for MDA-MB-436 and *p* < 0.001 for SUM-159, Fig. 2a and Supplementary Fig. 3A). Because IL-6 and IL-8 are often reported in tandem to promote TNBC growth, metastasis, and chemoresistance^21,30,31^, we also evaluated if IL-1A neutralization diminished docetaxel mediated production of IL-8. Indeed, IL-1A neutralization resulted in 66–73% reduction of IL-8 in the MSL TNBC cell lines (*p* < 0.01 for MDA-MB-436 and *p* < 0.001 for SUM-159, Fig. 2a and Supplementary Fig. 3A). We have previously shown that BL1 TNBCs do not produce IL-1A (Fig. 1e). We, therefore, investigated whether BL1 TNBCs are capable of downstream IL-1 signaling by treating two representative BL1 TNBC cell lines, MDA-MB-468 and HCC38, with recombinant human IL-1A. Treatment of BL1 TNBCs with recombinant human IL-1A resulted in significant production of IL-6 and IL-8 (Fig. 2b and Supplementary Fig. 3B) indicating that BL1 TNBCs are capable of downstream IL-1 signaling and only lacking in endogenous production of IL-1.

Next, we and others have previously shown that taxane therapy induces endoplasmic reticulum (ER) stress^32–34^. Surprisingly, docetaxel treatment increased the ER stress sensor IRE1α in MSL TNBCs, whereas BL1 TNBCs had no change after therapy (Fig. 2c, d and Supplementary Fig. 3C). Due to inconsistent findings with phosphorylated IRE1α between MSL and BL1 TNBCs, we verified docetaxel mediated inflammation was downstream of IRE1 with Kira6. Kira6 is an established IRE1 inhibitor^35,36^, and we found consistent abrogation of docetaxel induced IL-1A and IL-6 by Kira6 in both MSL TNBCs (Supplementary Fig. 3D). Furthermore, ER stress has been well-characterized for activation of MAPK pathway^37–39^. In alignment with previous reports, we observed that docetaxel treatment of MSL TNBC showed a correlative increase in both ERK1/2 phosphorylation and IL-1A (Fig. 2e). Cumulatively, our data show that docetaxel induces ER stress and inflammation in MSL TNBCs which is not observed in BL1 TNBCs.

**Fig. 3:**
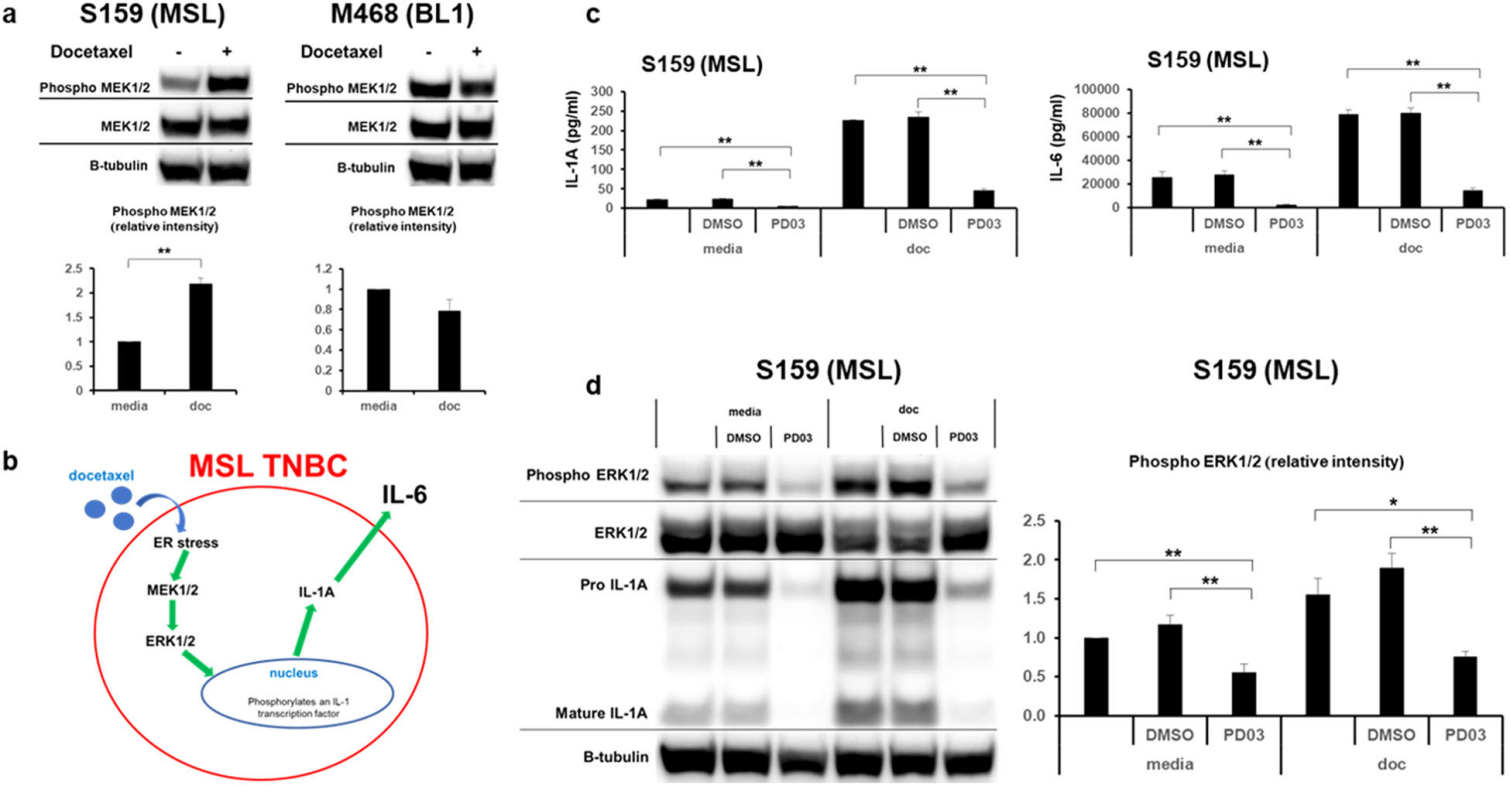
Docetaxel induced MAPK activity promotes autocrine IL-1A/IL-6 production in MSL TNBCs. **a** TNBCs were treated in the presence or absence of docetaxel (doc) at 4 ng/ml for 48 h. WB from whole-cell lysates was performed, shown are representative images. Average ± SEM densitometry analysis is from *n* = 3 biological replicates. **b** Cartoon representation of MAPK mediated induction of IL-1A/IL-6. **c** PD 0325901 (PD03) is a MEK inhibitor. MSL TNBC was treated for 48 h at the indicated conditions (doc = 4 ng/ml and PD03 = 1 μM), supernatants were measured for IL-1A and IL-6. Shown are average ± SEM ELISA results from *n* = 4 biological replicates. **d** WB from paired whole-cell lysates to supernatant samples from **c**, shown is a representative image. Average ± SEM densitometry analysis is from *n* = 4. Statistical analysis performed with unpaired *t*-test: *p < 0.05; **p < 0.01. All indicated experiments are representative of at least two independent experiments.

To investigate the potential relevance of asymmetrical MAPK pathway activation in the two TNBC subtypes, we found docetaxel treatment resulted in the phosphorylation of MEK1/2 in MSL cell lines SUM-159 and MDA-MB-436 (Fig. 3a and Supplementary Fig. 4A), whereas docetaxel did not activate MEK1/2 in BL1 cell lines MDA-MB-468 and HCC38. To confirm the relevance of the MAPK pathway, MSL TNBC cell lines were treated in the presence or absence of docetaxel with MEK inhibitor PD 0325901^40^. MEK inhibition led to a statistically significant reduction of IL-1A both at baseline levels (*p* < 0.001) and after docetaxel treatment (*p* < 0.001, Fig. 3c and Supplementary Fig. 4B) in both MSL TNBC cell lines. Furthermore, the reduction of IL-1A likewise led to decreased production of the downstream cytokine IL-6 in SUM-159 (*p* < 0.001, Fig. 3c). We next investigated the phosphorylation of downstream targets ERK1/2 to validate that the effect of PD 0325901 was the result of MEK inhibition^41^. We confirmed that PD 0325901 significantly reduced ERK1/2 phosphorylation both in the absence and presence of docetaxel (*p* < 0.01, Fig. 3d and Supplementary Fig. 4C) for both MSL TNBC cell lines. In addition, we also determined that MEK inhibition resulted in a clear reduction of pro-IL-1A (Fig. 3d and Supplementary Fig. 4C) which aligned with the extracellular IL-1A reduction results as in Fig. 3c and Supplementary Fig. 4B. These results indicate that docetaxel mediates activation of the MAPK pathway to initiate the IL-1A driven inflammation in MSL TNBCs (Fig. 3b). This mechanism does not occur in BL1 TNBCs as the MAPK pathway remains unchanged following docetaxel treatment. Furthermore, we found that MSL TNBCs responded dose-dependently to MEK inhibition by PD 0325901 (Supplementary Fig. 5A). Cumulatively, our data aligns with previous reports that MAPK pathway activates transcription factors that bind to the IL-1A promoter in tumorigenesis^42^. As IL-1A can be produced through MEK independent pathways^43,44^, we investigated the role of other reported pathways that can induce IL-1A. Inhibition of AP-1 resulted in no reduction of IL-1A and IL-6 in MSL TNBC cell lines (Supplementary Fig. 5B), whereas inhibition of NF-kB resulted in inconsistent reduction of IL-6 in MSL TNBC cell lines (Supplementary Fig. 5C). This suggests that NF-kB does not contribute to the MAPK/IL-1A axis, yet may play a minor independent role in IL-6 production. Cumulatively, these results show that docetaxel activates the MAPK pathway in MSL TNBCs which triggers an autocrine IL-1A cascade for subsequent inflammation. This mechanism is virtually absent in BL1 TNBCs as docetaxel treatment results in no MAPK activation.

### Docetaxel synergizes with IL-6 neutralization against MSL TNBCs in vitro

**Fig. 4:**
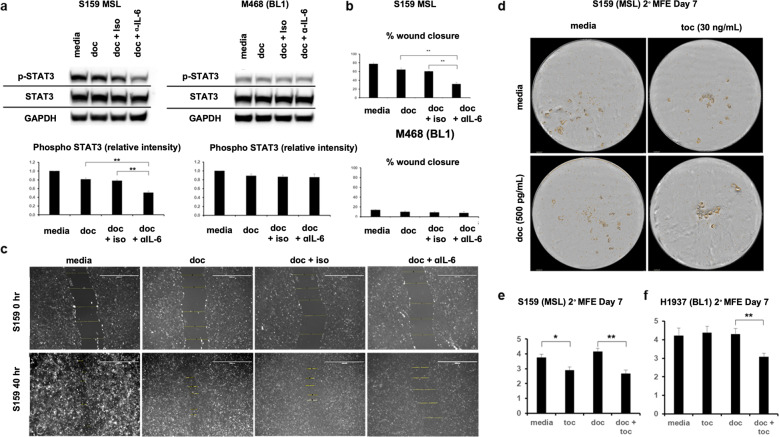
IL-6 neutralization in combination with docetaxel provides benefit against MSL TNBCs, but not BL1 TNBCs. **a** The indicated TNBC cell lines were treated with docetaxel (4 ng/ml) with or without anti IL-6 neutralizing antibody (0.1 μg/ml) or isotype control for 24 h, and then western blot was performed. Shown are representative western blot images and average ± SEM densitometry of phosphorylated STAT3 from *n* = 3 biological replicates for both TNBC cell lines. **b** Scratch migration for TNBCs treated for 40–48 h with or without docetaxel (4 ng/ml), anti IL-6 neutralizing antibody (1 μg/ml) and isotype control (1 μg/ml). Shown are average ± SEM % wound closure from *n* = 5 biological replicates for all cell lines. **c** Representative (from **b**) scratch migration image for S159 at *t* = 0 h and *t* = 40 h. **d** Secondary mammospheres treated in the presence or absence of docetaxel (500 pg/ml) or tocilizumab (30 ng/ml). Mammospheres were counted on day 7 on Incucyte by threshold of cell area >1256 μm^2^. Shown is a representative image from S159 (MSL) on day 7. **e** Shown are average ± SEM mammosphere formation efficiency (MFE) from *n* = 8 biological replicates of S159 (MSL). **f** Shown are average ± SEM MFE from *n* = 8 biological replicates of BL1 TNBC. Statistical analysis performed with unpaired *t*-test: *p < 0.05; **p < 0.01. All indicated experiments are representative of at least two independent experiments.

As IL-6 has a multi-factorial role in promoting cancer cell proliferation, metastasis, and cancer stem cell induction^19–23^, we investigated in vitro the potential efficacy of neutralizing MSL TNBC autocrine IL-6 production in combination with docetaxel treatment. Dual treatment of MSL TNBC cell lines with docetaxel and an IL-6 neutralizing antibody led to a significant reduction in phosphorylation of STAT3, a potent transcription factor for cancer proliferation (Fig. 4a and Supplementary Fig. 6A). In contrast, IL-6 inhibition resulted in inconsistent reduction of STAT3 phosphorylation in BL1 TNBCs. We then determined whether IL-6 neutralization reduced TNBC cell migratory capability. Scratch migration assays showed that docetaxel in combination with IL-6 neutralizing antibody led to a significant decrease in wound closure in MSL TNBC cell lines and not BL1 TNBCs (Fig. 4b, c and Supplementary Fig. 6B). Tocilizumab is a humanized anti-IL6R antibody with FDA approval for arthritis but not yet for any cancer immunotherapy applications. As mammospheres are used as an in vitro surrogate for cancer stem cells^45^, we investigated the efficacy of tocilizumab in abrogating mammosphere formation efficiency (MFE). MSL cell line SUM-159 had diminished secondary mammosphere colonies when co-treated with docetaxel and tocilizumab compared to chemotherapy alone (Fig. 4d). Although mono-therapy of tocilizumab resulted in decreased mammospheres in only one MSL TNBC cell line, combination therapy of docetaxel and tocilizumab led to a significant reduction of MFE in both MSL cell lines compared to docetaxel treatment alone (Fig. 4e and Supplementary Fig. 6C). Surprisingly, docetaxel combined with tocilizumab also led to a significant reduction of MFE in a BL1 TNBC cell line (Fig. 4f). Despite these unexpected results, our cumulative data clearly shows a consistent benefit of IL-6 targeted therapy against MSL TNBCs compared to inconsistent benefit against BL1 TNBCs in vitro.

### Tocilizumab combined with docetaxel has efficacy against MSL and not BL1 xenografts

**Fig. 5:**
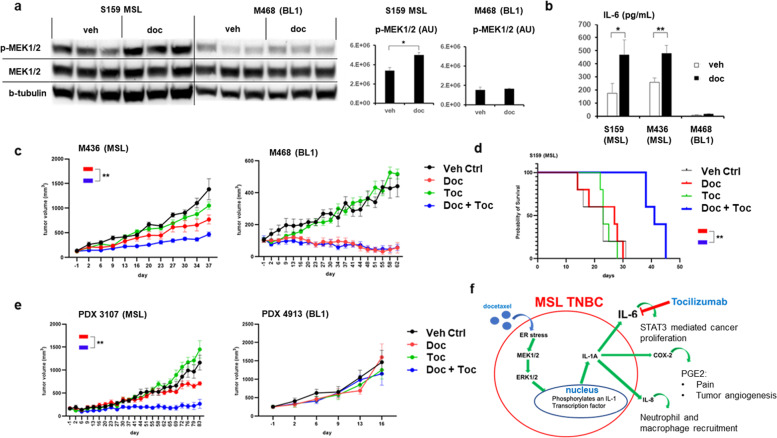
Tocilizumab in combination with docetaxel provides benefit against MSL TNBCs in vivo, and not against BL1 TNBCs. **a** Female NSGs were implanted with human TNBC cell lines and treated for 2 days with vehicle (veh) or docetaxel (doc) 20 mg/kg. WB was performed from primary tumor and shown are average ± SEM densitometry (*n* = 3 pairs for both cell line xenografts). The six indicated MSL samples were performed on one membrane and the six BL1 samples were performed on a separate membrane. **b** Female NSGs were implanted with human TNBC cell lines and treated with veh or doc (20 mg/kg). Two days later, serum was collected and human IL-6 average ± SEM was determined by ELISA. S159 = 10 pairs, M436 = 8 pairs, M468 = 6 pairs. **a**, **b** Significance was determined by unpaired *t*-test. **c** Shown are average tumor volumes ± SEM from cell line xenograft studies. M436 animals (*n* = 6 per group) received five cycles of therapy. M468 animals (*n* = 6 per group) received four cycles of therapy, however cycle 2 lacked any tocilizumab treatment for applicable groups. Tumor volume significance was determined by 2-way ANOVA. **d** Kaplan–Meier (KM) survival from S159 xenografts (*n* = 5 per group), animals received four cycles of therapy. **e** Shown are average tumor volumes ± SEM from PDX studies. 4913 animals (*n* = 5 per group) received two cycles of therapy. 3107 animals (*n* = 6 per group) received six cycles of therapy. Tumor volume significance was determined by mixed effects analysis. **f** Model figure of MSL TNBC resisting chemotherapy mediated by MAPK induction of IL-1A/IL-6. For all statistical analysis: *p < 0.05; **p < 0.01.

To validate the relevance of docetaxel induced MAPK activity in vivo, we implanted cell line xenografts into NSG female mice. Concurrent with our in vitro data, docetaxel treatment led to significant phosphorylation of MEK1/2 in MSL xenografts and not BL1 xenografts (Fig. 5a). Furthermore, docetaxel increased human IL-6 production in mice bearing MSL TNBC xenografts and not BL1 xenografts (Fig. 5b). Although we found inconsistent induction of IL-1A in MSL xenografts, we confirmed virtually no detectable IL-1A in BL1 xenografts (Supplementary Fig. 7A, B). Next, to investigate the efficacy of tocilizumab in vivo, we distributed mice bearing MSL or BL1 xenografts into groups receiving mono-therapy of docetaxel, tocilizumab, or dual therapy. MDA-MB-436 (MSL) bearing animals had a statistically significant delayed tumor growth when receiving dual therapy compared to chemotherapy alone (*p* < 0.001), whereas dual therapy provided no benefit for MDA-MB-468 (BL1) bearing animals (Fig. 5c). Furthermore, in a second MSL (S159) xenograft model, animals receiving docetaxel in combination with tocilizumab had a median increase in survival of 2 weeks (*p* < 0.01) compared to animals receiving chemotherapy alone (Fig. 5d). Our lab has previously characterized TNBC patient-derived xenografts (PDX) as MSL and BL1 subtypes^46^. We, therefore, investigated the efficacy of our anti-inflammatory regimen in mice bearing MSL and BL1 PDXs. Similar to our cell line xenografts data, animals bearing MSL PDX benefited when receiving docetaxel with tocilizumab (*p* < 0.001) as compared to chemotherapy alone (Fig. 5e) and the benefit of dual therapy did not occur for animals bearing BL1 PDX. Although host murine IL-6 will not bind to human IL-6R^47^, we validated that docetaxel induction of IL-6 was unique for a TNBC subtype rather than a broad cellular response to chemotherapy. Both immunocompromised NSG and immunocompetent C57BL/6 animals had no statistical induction of mouse IL-6 after chemotherapy (Supplementary Fig. 7C).

## Discussion

TNBC patients currently lack specific targeted therapeutics and have shown marginal benefit to immune checkpoint blockade therapy. It has previously been shown by others that high expression of IL-1A and IL-6 both correlate with poor prognosis for breast cancer patients^21,42^. Other preclinical studies have investigated the efficacy of tocilizumab against breast cancers broadly without necessarily delineating which TNBC patients would benefit the most from tocilizumab^48–50^. Here, we provide evidence that a molecular subset of TNBC resists conventional chemotherapy by a MAPK mediated autocrine IL-1A/IL-6 axis (Fig. 5f). Furthermore, our data potentially identifies a population of TNBC patients that may benefit from supplementing chemotherapy with tocilizumab to eliminate autocrine tumor production of IL-6. Currently, tocilizumab is not FDA approved for any tumor malignancies. Previous clinical trials have found that targeted IL-6 therapies provided no benefit in hematological malignancies, renal, and prostate cancer^51,52^. To the best of our knowledge, no clinical trial has completed an investigation of the potential efficacy of tocilizumab for TNBC patients.

We hypothesize that differential benefit from IL-6 blockade may be due to a gradient physiological effect of IL-6. BL1 TNBCs produce quantifiable levels of IL-6 both in vitro and in vivo, but to a lesser extent compared to MSL TNBCs. Neutralization of BL1 autocrine IL-6 provided no benefit in reducing tumor cell proliferation in vivo. In contrast, MSL TNBCs producing substantial IL-6 were effectively targeted with tocilizumab possibly providing insight that a gradient effect from autocrine IL-6 is necessary to promote tumor resistance. Therefore, our model demonstrates that tocilizumab will not be beneficial universally for TNBC patients. This novel therapeutic regimen will likely only improve the outcome for patients with chemoresistant tumors driven by MAPK induced IL-6.

This study identified MAPK activity-induced expression of other inflammatory mediators that may also be candidates for targeted therapy against MSL tumors. Although COX-2 has been well characterized to promote tumor angiogenesis and downregulate anti-tumor immunity, TNBC clinical trials targeting COX-2 have been largely unsuccessful^53–55^. Our data shows that only a subset of TNBCs undergo MAPK induced expression of COX-2 after docetaxel therapy, which may indicate that COX-2 inhibitors may not provide benefit for all TNBC patients. We, therefore, hypothesize that upon subtyping TNBC patients and performing a retrospective analysis of completed clinical trials investigating COX-2 inhibitors, there may be a differential response rate to COX-2 inhibitors based on tumor subtypes and MAPK activity of patient tumors.

IL-8 is a chemokine that recruits macrophages and neutrophils into the tumor microenvironment, and tumor-associated macrophages and neutrophils have been demonstrated to favor a pro-tumor niche^56^. We identified IL-8 as another downstream inflammatory mediator produced by docetaxel resistant MAPK active tumor cells. IL-8 anti-biologic therapy has already been established to be safe in phase I trials against metastatic tumors and is currently under investigation in several phase II studies^57^. Cumulatively, we endeavor to provide a rationale for a clinical trial that identifies TNBC patients with chemo-refractory tumors driven by MAPK activity. In essence, these patients may benefit from supplementing conventional chemotherapy with targeted therapies against MAPK-driven production of IL-6, COX-2, and IL-8.

The chief limitation of our study is the absence of immune cellular contribution in the context of IL-6 blockade in vivo. Although we acknowledge that IL-6 has a broad cellular effect and is produced by diverse cell types, we provide clear evidence that docetaxel mediated MAPK/IL-6 activity is specific for a subtype of TNBCs. This is evident as docetaxel failed to induce host mouse IL-6 in both immunocompromised and immune-competent animals. Finally, tocilizumab will neutralize IL-6 signaling by antagonizing IL-6R regardless of the cellular source of IL-6 in the tumor microenvironment.

It should be noted that while we have consistently completed two representative MSL cell lines vs. two representative BL1 cell lines (i.e., S159 and M436 vs. M468 and H38), there were two notable exceptions: 1. Scratch migration (Supplementary Fig. 6B), M436 did not migrate during the indicated time conditions. We, therefore, substituted with M231 as a second MSL TNBC for scratch migration. 2. Mammospheres (Fig. 4f), we failed to initiate mammospheres with M468 after 2 months. Published literature confirmed that M468 does not form mammospheres. We could not find evidence that others established successful H38 mammospheres. H1937 was the only BL1 TNBC cell line that others established mammospheres; we, therefore, investigated only H1937 as the sole BL1 cell line for mammospheres.

Although BL1 TNBCs can produce autocrine IL-6, tocilizumab provides no benefit against two BL1 xenografts in vivo. Due to inconsistent benefit of neutralizing IL-6 against BL1 tumors in vitro (Fig. 4f and Supplementary Fig. 6A), we acknowledge that tocilizumab may possibly provide benefit for some non-MSL TNBC patients. However, our principal hypothesis is that tocilizumab therapy may provide greater efficacy against tumors with MAPK-driven IL-6 production. To investigate our hypothesis, we would design a phase 2 clinical trial screening 100–200 TNBC patients by RNA sequencing for sufficient enrollment of MSL patients as MSL is representative of 10–19% of TNBC patients^8,9^. All enrolled MSL trial patients would receive taxane therapy in combination with tocilizumab and evaluated for regimen efficacy. In addition, baseline tissue analysis for MAPK expression between responders and non-responders will be evaluated as potential predictive biomarkers for tocilizumab therapy.

Our investigation potentially identifies a population of TNBC patients that may benefit from supplementing conventional chemotherapy with a novel anti-inflammatory cocktail regimen to negate MAPK-driven autocrine cytokines. In addition, our results may also apply to other cancer pathologies reliant on MAPK pathway for therapy resistance. Ultimately, it is a paramount objective to improve the identification of cancer patients for targeted therapy regimens.

## Methods

### Cell culture, reagents, and antibodies

The following human MSL and BL1 TNBC cell lines were used for this study^7^: SUM-159, MDA-MB-436, MDA-MB-231, MDA-MB-157, MDA-MB-468, HCC38, HCC1937, and HCC1599. Cell lines were purchased from ATCC, authenticated, and regularly tested for mycoplasma. All cell lines were cultured in DMEM (HyClone- Logan, Utah) supplemented with 10% fetal bovine serum (FBS), Antibiotic-Antimycotic (GenDepot- Katy, Texas), and L-glutamine (Corning- Manassas, Virginia) in a 5% CO_2_ incubator at 37 °C. For the indicated in vitro conditions, the following tissue culture reagents were used at following concentrations unless noted differently: docetaxel (NovaPlus, 4 ng/ml from Ebewe Pharma, Austria), human IL-1A neutralizing antibody (R&D clone 4414, 1 μg/ml), mouse IgG_2A_ isotype control antibody (R&D clone 20102, 1 μg/ml), recombinant human IL-1A (R&D 200-LA, 2 ng/ml), DMSO (Sigma D2650), PD 0325901 (R&D 4192, 1 μM), human IL-6 neutralizing antibody (BD Biosciences clone MQ2-13A5, 0.1 μg/ml), rat IgG_1_ isotype control antibody (BD Biosciences clone R3-34, 0.1 μg/ml), tocilizumab (Roche, 30 ng/ml from San Francisco, CA), and Kira6 (Cayman Chemical 19151 from Ann Arbor, MI).

Anti-human antibodies used for ELISAs: IL-1A capture and detection (R&D clone 4414 and catalog# BAF200, respectively), IL-6 capture and detection (BD Biosciences clones MQ2-13A5 and MQ2-39C3, respectively), IL-8 capture and detection (BD Biosciences clones G265-5 and G265-8, respectively). R&D items from Minneapolis, MN; Sigma items from St. Louis, MO; BD Biosciences items from San Jose, Ca.

The following Cell Signaling Technology (Danvers, MA) anti-human antibodies were used for western blots: COX2 (12282), β-actin (4970), GP130 (3732), β-tubulin (2146), ERK1/2 (4695), phospho-ERK1/2 (9101), MEK1/2 (4694), phospho-MEK1/2 (9154), GAPDH (5174), STAT3 (9139), IRE1α (3294), and phospho-STAT3 (9145). Abcam (Cambridge, MA) anti-human IL-1A (ab206410), anti-human phosphor-IRE1 (ab124945) and anti-human IL-6R (ab128008) were also used for western blots.

### RNA sequencing analysis

Human TNBC cell lines were treated in the presence or absence of docetaxel (4 ng/ml) for 48 h and RNA was extracted with RNeasy Plus Mini Kit (Qiagen from Hilden, Germany). Genomic DNA was removed with gDNA eliminator spin columns and total RNA purification was done according to manufacturer’s instructions. cDNA library preparation and RNA Sequencing was performed by the Genome Sequencing Facility of Greehey Children’s Cancer Research Institute at the University of Texas Health San Antonio as previously described^58^. RNA sequencing data processing and analysis of RPKM values were done as previously described^59^. In brief, RNAseq reads were quality filtered using Trimmomatic (10.1093/bioinformatics/btu170) and aligned to the human reference genome assembly GRCh38.p12 using HISAT2. Next, HTSeq was used to determine how many reads mapped to each features (10.1093/bioinformatics/btu638). Differential gene expression was performed using a permutation test for linear models. A total of 921 out of 24850 ENTREZ gene ID passed a fold-change cutoff of 4 and a *p*-value cutoff of 0.05 for pathway analysis on InnateDB (10.1093/nar/gks1147). Pathway over-representation analysis was performed using hypergeometric distribution test and Benjamini–Hochberg false discovery rate correction.

### Ingenuity Pathway Analysis (IPA)

One RNA sequencing dataset analyzed fold change ratio of averaged RPKM gene expression from untreated MSL TNBCs compared to averaged RPKM gene expression from untreated BL1 TNBCs. The other dataset analyzed fold change ratio of averaged RPKM gene expression from docetaxel-treated MSL TNBCs compared to averaged RPKM gene expression from docetaxel-treated BL1 TNBCs. Fold change ratio cut-off of 5 or −5 was utilized for both datasets and the “Core Analysis” function on IPA was utilized for upstream analysis of uploaded datasets. Estimated RPKM values were used to visualize heatmaps.

### Western blot analysis

Human TNBC cell lines were treated under the indicated conditions and whole-cell lysates were collected with RIPA buffer (Sigma), 1% protease inhibitor, and 1% phosphatase inhibitor. In brief, cells were washed with cold phosphate-buffered saline (PBS) twice and lysed with complete RIPA buffer. Culture wells were then scraped with cell scrapers, lysate solutions were collected and incubated on ice for 20 min. Following centrifugation at 4 °C max speed for 10 min, whole-cell lysates were collected for western blot. Protein gel electrophoresis was performed on Bolt™ 4–12% Bis-Tris Plus Gels (ThermoFisher- Carlsbad, CA) followed by transfer onto 0.2 μm PVDF membranes. After 1 hr blocking in 5% milk, blots were stained overnight with previously listed primary antibodies at 4 °C in 5% BSA.

All primary antibodies for western blot (WB) were diluted 1:1000, except anti-human IL-6R (ab128008) was diluted at 1:500. Following overnight incubation, blots were developed with Cell Signaling Technology secondary antibodies anti-rabbit IgG HRP-linked (#7074, 1:2000 dilution) or anti-mouse IgG HRP-linked (#7076, 1:2000 dilution). Blot signals were detected with the BioRad XRS+ and densitometry analysis was performed with BioRad’s Image Lab software. After development of phosphorylated targets, blots were stripped with Restore™ Western Blot Stripping Buffer (ThermoFisher) and re-probed with relevant total protein antibody. All blots derive from the same experiment and were processed in parallel, except Figs. 1f, 5a and Supplementary Fig. 7A (see individual figure legends for more details).

### ELISA analysis

Cultured supernatants from TNBC cell lines treated under indicated conditions were collected after 48 h. Human IL-1A, IL-6, and IL-8 ELISAs were performed with previously listed paired capture and detection antibodies according to the manufacturer’s instructions. Human prostaglandin E2 (PGE2) ELISA was performed according to the manufacturer’s instructions (Cayman 514010- Ann Arbor, MI).

For in vivo analysis of human IL-6 in mice implanted with human cell lines, serum was collected after 2 days of treatment with vehicle or docetaxel. Mouse sera were analyzed with the previously listed human-specific ELISA antibodies for human TNBC produced IL-6 in vivo.

### RT-PCR

Human TNBC cell lines were cultured in the presence or absence of docetaxel (4 ng/ml) for 48 h, and RNA was isolated with Qiagen RNeasy Mini Kit (74104) according to manufacturer’s instructions. cDNA synthesis was performed with BioRad’s iScript cDNA synthesis kit (1708891) and real-time PCR (RT-PCR) was performed with BioRad’s iQ SYBR Green Supermix (1708882) following the manufacturer’s instructions on BioRad’s CFX96. BioRad items are from Hercules, CA. Primers are listed under Supplementary Table 1.

### Mammosphere formation efficiency

TNBC cell lines were cultured as primary mammospheres with MammoCult medium (Stem Cell Tech #05620) supplemented with heparin (Stem Cell Tech #07980) and hydrocortisone (Stem Cell Tech # 07925) according to manufacturer’s instructions. Stem Cell Tech items are from Cambridge, MA. Cells were seeded at 24,000 cells per well in 2 mL of complete MammoCult medium in six-well ultra-low attachment plates. Every 2–3 days, 1 mL of fresh MammoCult medium was added to the wells. After 8–11 days, cells were collected with 0.05% trypsin and neutralized with 10% FBS for secondary mammosphere assays. The cells were then re-suspended in complete MammoCult medium and seeded at 8000 cells per well in 24-well ultra-low attachment plates. Secondary mammospheres were treated under the presence or absence of docetaxel (500 pg/ml) and tocilizumab (30 ng/ml) on day zero. On day 3, cells were re-fed with complete MammoCult medium and tocilizumab (30 ng/ml final) was re-added to applicable wells. On day 7, mammospheres were quantified with Incucyte Live-Cell Imaging System and its bundled software. Quantification included a minimum cell area of 1256 μm^2^ and MFE was calculated as follows: (number of spheres/8000) × 100%. Mammosphere assays were repeated with eight replicates for each treatment group.

### Scratch migration assay

TNBC cell lines were seeded overnight in six-well culture plates and grown to 60–90% confluence. The cell monolayer was then scratched with a p200 pipette tip, and the monolayer was washed with PBS. The monolayer was then left untreated or treated with docetaxel (4 ng/ml) in the presence or absence of IL-6 neutralizing antibody or isotype control antibody (1 μg/ml). The scratch widths were then captured on the EVOS brightfield microscope (ThermoFisher) at ×2 magnification for time = 0 h. Cells were incubated for 40–48 h, and then the scratch widths were re-captured at ×2 magnification. At all time points, average scratch widths were determined from *n* = 5 measurements. Percent wound closure was calculated as follows: 100 × [(scratch width at *T* = 0 h) − (scratch width at *T* = final h)]/(scratch width at *T* = 0 h).

### Immunofluorescence

SUM-159 cell lines were cultured overnight in eight chambered cell culture slides (Corning 354118) pre-coated with Poly-L-lysine (Sigma P1399). Cells were then treated in the presence or absence of docetaxel (4 ng/ml) for 48 h and then stained for immunofluorescence. In brief, cells were fixed with 4% paraformaldehyde and permeabilized with 100% methanol. After blocking, cells were stained for Phospho ERK1/2 (R&D MAB1018) overnight at 4 C. The next day, cells were washed and stained with goat anti-rabbit Alexa Fluor 546 (ThermoFisher A-11035). Afterwards, cells were washed and stained for IL-1A (R&D MAB200) followed by goat anti-mouse Alexa Fluor 488 (ThermoFisher A-11001). Slides were mounted with Vectashield (Vector Labs H-1200 from Burlingame, Ca) and sealed with coverslips. Slides were imaged on the EVOS FL Auto Imaging System.

### In vivo experiments

The Houston Methodist Hospital Research Institute Animal Care and Use Review Office approved this study. All tumor models were developed orthotopically in the mammary fat pad of female NOD scid gamma (NSG) mice and handled as described previously^58^. In brief, cell lines were injected into the right mammary fat pad of female NSGs: SUM-159 (3 × 10^6^), MDA-MB-436 (9 × 10^6^), and MDA-MB-468 (2 × 10^6^). PDX were derived and subtyped as MSL and BL1 as previously described^46,58^. After tumors were grown orthotopically to 100–200 mm^3^ in volume, mice were randomized into groups of (i) vehicle control, (ii) docetaxel (20 mg/kg), (iii) tocilizumab (20 mg/kg), (iv) docetaxel and tocilizumab (both 20 mg/kg). All groups were treated intraperitoneally once every 2 weeks.

For in vivo analysis of murine IL-6 production following docetaxel therapy, female NSG and C57BL/6 mice were treated with vehicle or docetaxel (20 mg/kg). Sera were collected after 2 days and analyzed with mouse IL-6 ELISA kit (BD Biosciences 550950).

### Statistical analysis

Statistical analyses were performed using two-tailed unpaired *t*-tests in Microsoft Excel. For all mice model experiments, outcomes of interest included tumor growth kinetics, biomarker expression and disease specific survival. Survival outcomes were compared using log-rank (Mantel–Cox) test and visualized using Kaplan–Meier curves. Two-way ANOVA and log-rank (Mantel–Cox) tests were performed using GraphPad Prism 8.

### Reporting summary

Further information on research design is available in the Nature Research Reporting Summary linked to this article.

## Supplementary information

Supplmental material

Reporting Summary

## Data Availability

The datasets supporting the conclusions for the current study are stored in a secured shared drive and will be shared by the corresponding author upon reasonable request. The raw sequencing files were uploaded to the Sequence Read Archive (SRA) database with the project number PRJNA767195. It will be released as manuscript is published.
