# Supplementary material for: Tocilizumab overcomes chemotherapy resistance in mesenchymal stem-like breast cancer by negating autocrine IL-1A induction of IL-6
Source: NPJ Breast Cancer. 2022 Mar 8;8:30. doi: 10.1038/s41523-021-00371-0 (PMC8904846; doi:10.1038/s41523-021-00371-0)

Tocilizumab overcomes chemotherapy resistance in mesenchymal stem-like breast cancer by negating autocrine IL-1A induction of IL-6

Andrew W. Chung[1,2,3], Anthony J. Kozielski[2,3], Wei Qian[2,3], Jianying Zhou[2,3], Ann C. Anselme[1,2,3], Alfred A. Chan[4], Ping-Ying Pan[2,3], Delphine J. Lee[4,5], Jenny C. Chang[2,3*]

[1]Texas A&M University Health Science Center, Bryan, TX 77807, USA

[2]Houston Methodist Research Institute, Houston, TX 77030, USA

[3]Houston Methodist Cancer Center, Houston, TX 77030, USA

[4]The Lundquist Institute, Torrance, CA 90502, USA

[5]David Geffen School of Medicine at Los Angeles, CA 90095, USA

Additional information:

* To whom correspondence should be addressed: 6445 Main St., P21-34, Houston, TX 77030, USA. Phone: 713-441-0681, Fax: 713-793-1642, E-mail: jcchang@houstonmethodist.org

Supplemental Materials:

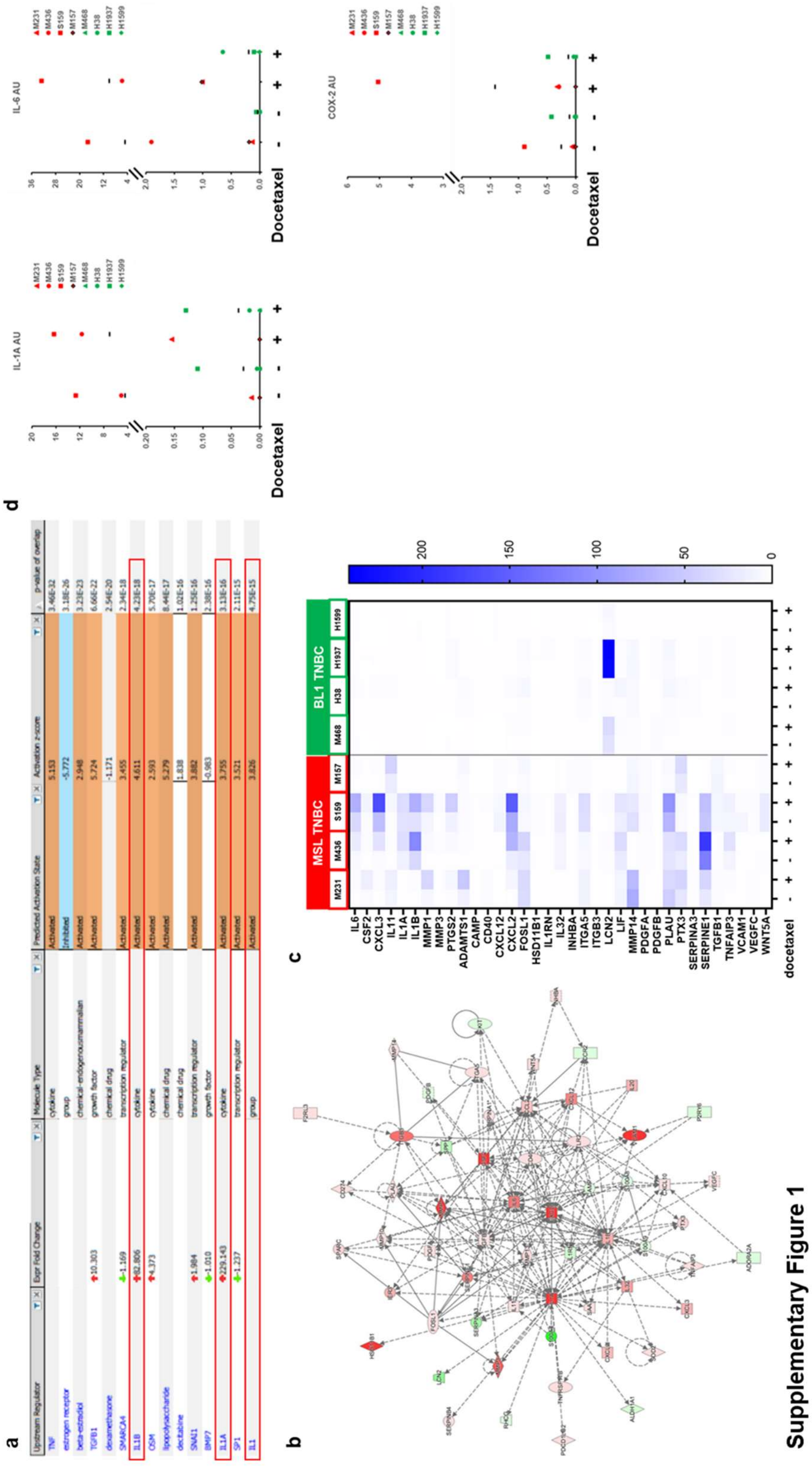

Supplementary Figure 1

Supplementary Figure 1. RNA sequencing predicts higher expression of immune molecules in MSL TNBCs compared to BL1 TNBCs. Four MSL TNBC and four BL1 TNBC cell lines were treated in presence or absence of docetaxel (4 ng/ml) for 48 hours and RNA sequencing was performed. A. IPA upstream regulator analysis of fold change ratio (cutoff value of 5 or -5) comparing averaged RPKM from four untreated MSL TNBC to averaged RPKM from four untreated BL1 TNBC. B. IPA pathway builder of IL1A upstream regulator network overlayed with dataset from Supplementary Fig. S1A (fold change ratio comparing averaged MSL untreated to averaged BL1 untreated samples). C. Heat map expression of RPKM values from selection of genes shown in IL1A upstream regulator network from **Fig. 1B**. D. Confirmatory qPCR for same samples used in RNA Seq experiment, shown are 2^-ΔCt values. Red represents MSL TNBC cell lines and green represents BL1 TNBC cell lines.

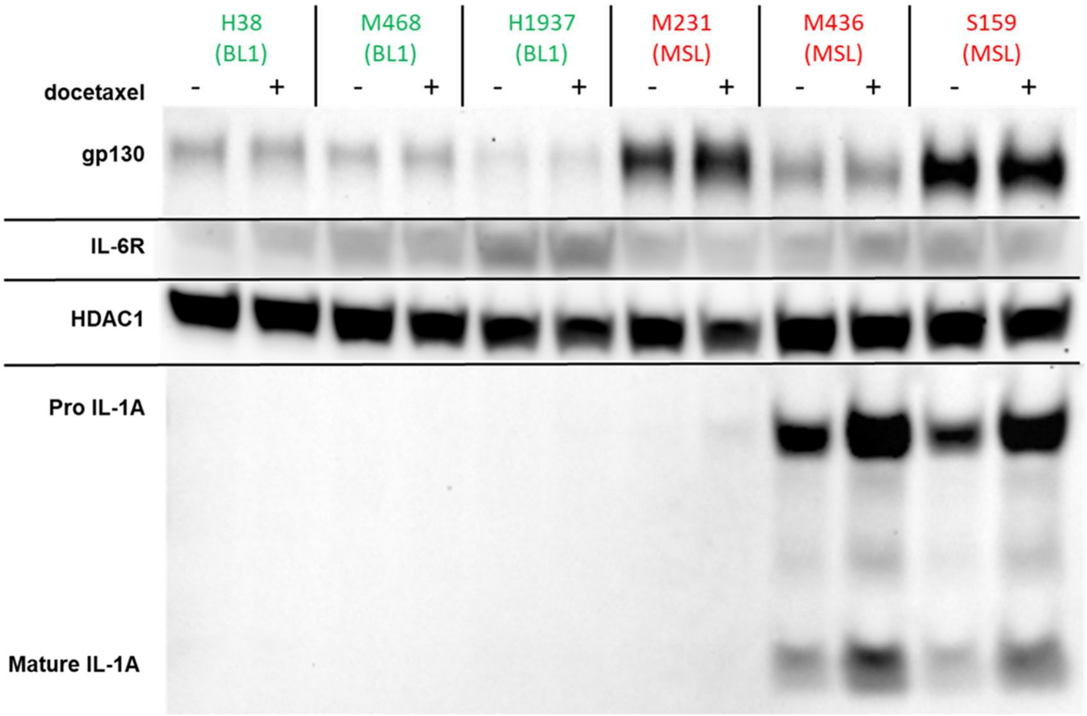

Supplementary Figure 2

Supplementary Figure 2. MSL TNBCs produce pro IL-1A in response to docetaxel, but not BL1 TNBCs. MSL TNBC cell lines and BL1 TNBC cell lines treated with or without docetaxel (4 ng/ml) for 48 hours, western blot was performed for indicated proteins.

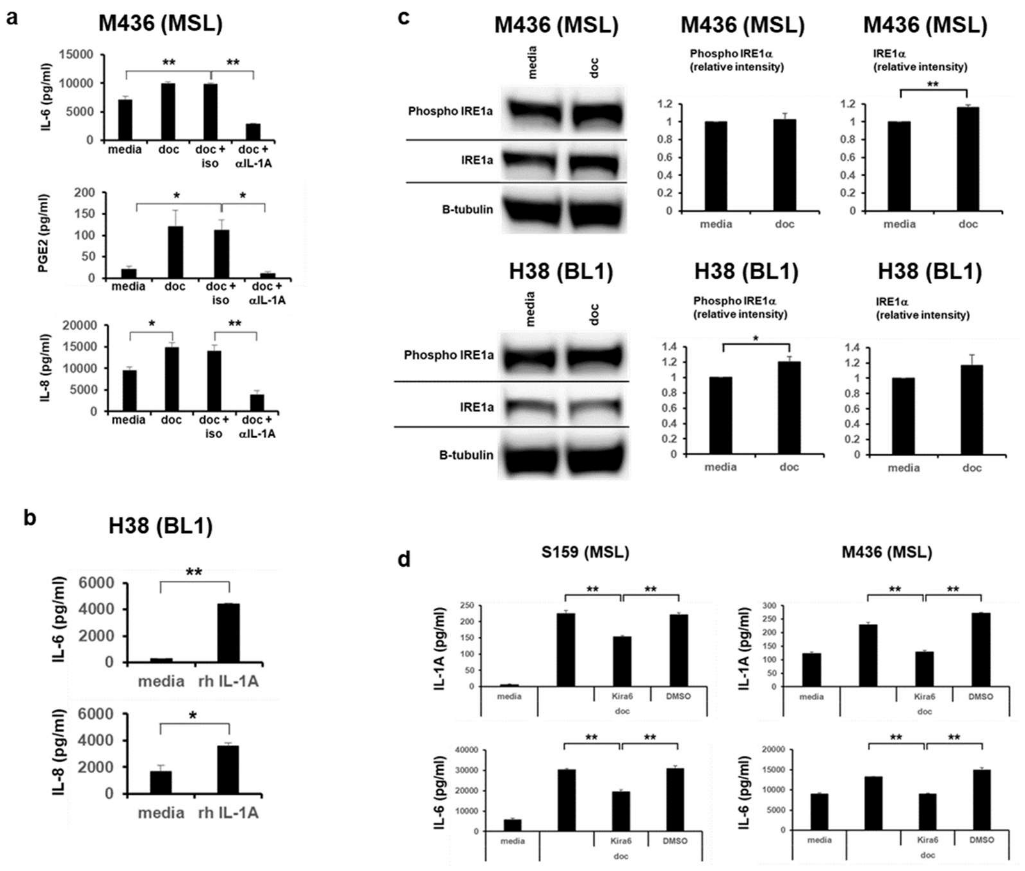

Supplementary Figure 3

Supplementary Figure 3. IL-1A is the upstream cytokine that promotes docetaxel mediated inflammation. A. Shown are IL-6, PGE2, and IL-8 ELISA results from supernatants collected 48 hrs from human TNBC cell line treated in presence or absence of docetaxel (doc) at 4ng/ml, NA/LE IL-1A mAb (1μg/ml) or NA/LE isotype mIgG2a (1μg/ml). B. BL1 TNBC treated in presence or absence of recombinant human (rh) IL-1A (2ng/ml) for 48 hrs and ELISAs analyzed supernatants for IL-6 and IL-8. Shown are average ± SEM from n=3 biological replicates. C. TNBCs were treated in the presence or absence of doc (4 ng/ml) for 48 hrs. WB from whole cell lysates was performed, shown are representative images. Average ± SEM densitometry analysis is from n=3. D. Kira6 is an IRE1 inhibitor. MSL TNBC was treated for 48 hrs at the indicated conditions (doc=4 ng/ml and Kira6=0.3μM) and supernatant was analyzed for IL-1A and IL-6. Shown are average ± SEM ELISA results representative from n=3 biological replicates. Statistical analysis performed with unpaired t-test: * $p < 0.05$; ** $p < 0.01$. All indicated experiments are representative of at least two independent experiments.

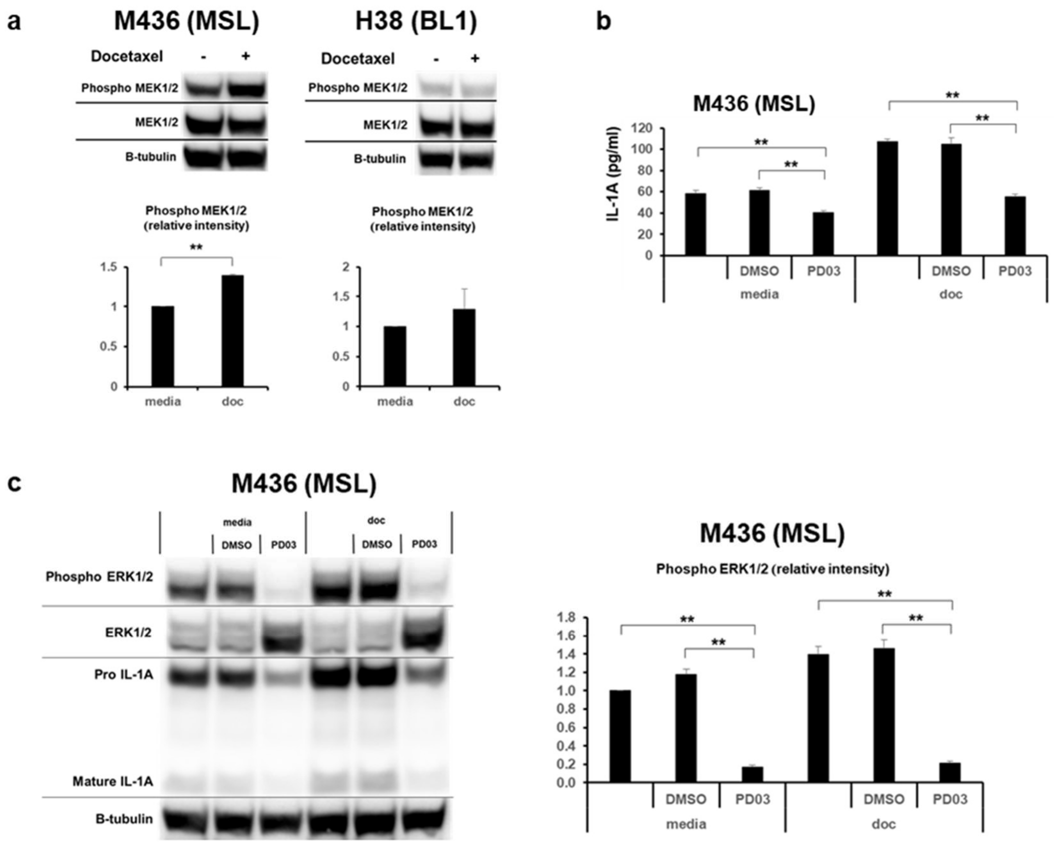

Supplementary Figure 4

Supplementary Figure 4. Docetaxel induced MAPK activity promotes autocrine IL-1A/IL-6 production in MSL TNBCs. A. Indicated cell lines were treated in the presence or absence of docetaxel (doc) at 4ng/ml for 48 hrs. WB from whole cell lysates was performed, shown are representative images. Average ± SEM densitometry analysis is from n=3 biological replicates. B. PD 0325901 (PD03) is a MEK inhibitor. MSL TNBC was treated for 48 hrs at the indicated conditions (doc=4 ng/ml and PD03=1μM) and supernatant was analyzed for IL-1A. Shown are average ± SEM ELISA results from n=4 biological replicates. C. WB from paired whole cell lysates as supernatant samples from Supplemental Fig. S4B, shown is a representative image. Average ± SEM densitometry analysis is from n=4. Statistical analysis performed with unpaired t-test: * $p < 0.05$; ** $p < 0.01$. All indicated experiments are representative of at least two independent experiments.

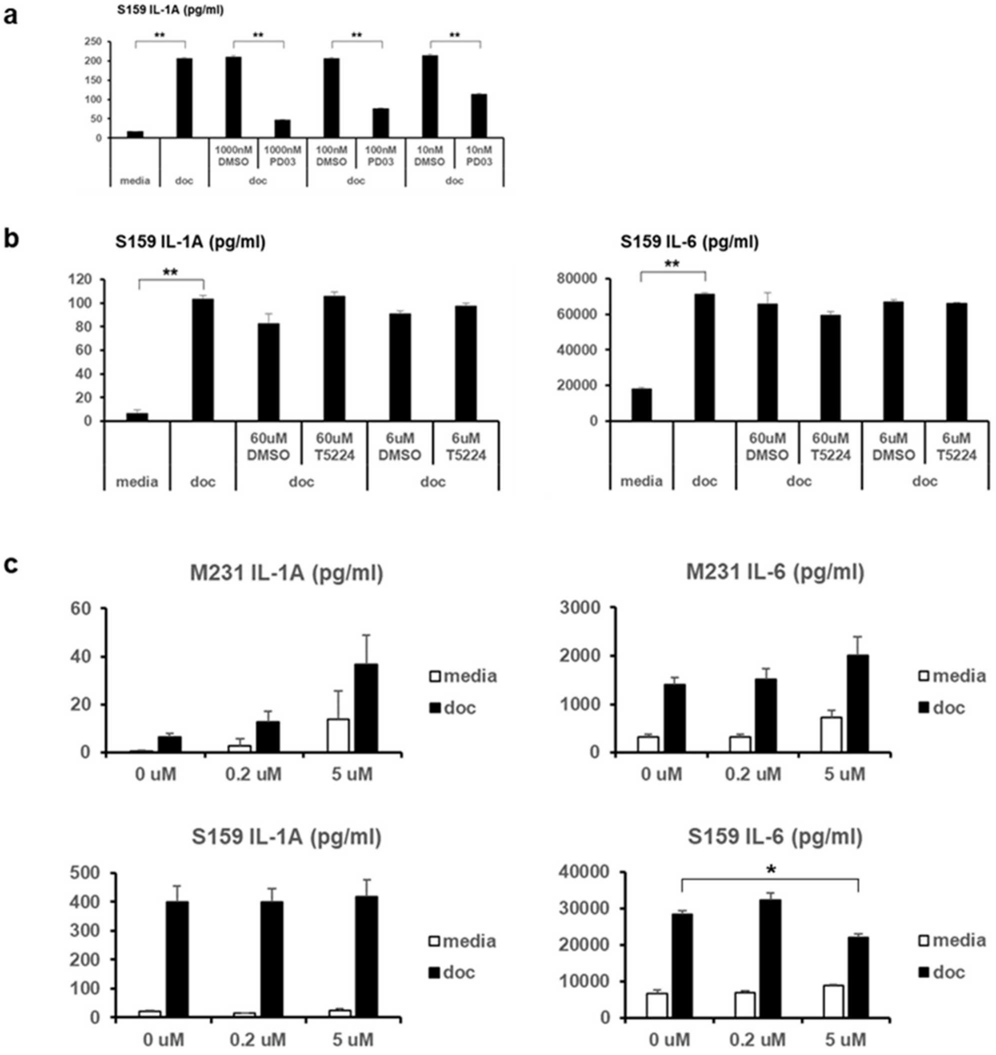

Supplementary Figure 5

Supplementary Figure 5. Dose dependent response of MEK1/2 inhibition against docetaxel treated MSL TNBCs. Shown are supernatant analysis from ELISA after 48 hour culture. MSL TNBC cell lines were treated with docetaxel (4 ng/ml) and the following inhibitors: A. PD03 (PD 0325901) is a MEK inhibitor and shown is average technical replicates ± SEM, representative from two independent experiments. B. T5224 is an AP-1 inhibitor and shown is average technical replicates ± SEM, representative from two independent experiments. C. SC75741 is a NFkB inhibitor and shown is average ± SEM from two independent experiments with n=3 biological replicates. Statistical analysis performed with unpaired t-test: * $p < 0.05$; ** $p < 0.01$.

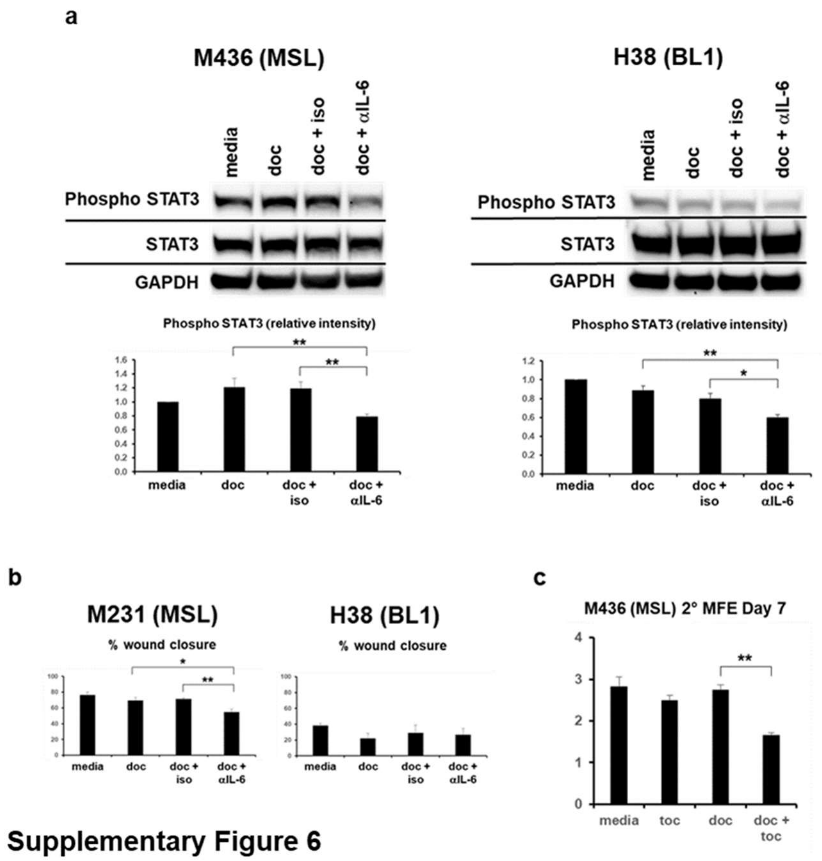

Supplementary Figure 6

Supplementary Figure 6. IL-6 neutralization in combination with docetaxel provides benefit against MSL TNBCs, but not BL1 TNBCs. A. Indicated TNBC lines were treated with docetaxel (4 ng/ml) with or without anti IL-6 neutralizing antibody (0.1 μg/ml) or isotype control for 24 hours, and then western blot was performed. Shown are representative western blot images and average ± SEM densitometry of phosphorylated STAT3 from n=3 biological replicates. B. Scratch migration for TNBCs treated for 40-48 hours with or without docetaxel (4 ng/ml), anti IL-6 neutralizing antibody (1 μg/ml) and isotype control (1 μg/ml). Shown are average ± SEM % wound closure from n=5 biological replicates for all cell lines. C. Secondary mammospheres treated in the presence or absence of docetaxel (500 pg/ml) or tocilizumab (30 ng/ml). Mammospheres were counted on day 7 on Incucyte by threshold of cell area > 1256 um^2. Shown are average ± SEM mammosphere formation efficiency (MFE) from n=8 biological replicates of M436 (MSL). Statistical analysis performed with unpaired t-test: * $p < 0.05$; ** $p < 0.01$. All indicated experiments are representative of at least two independent experiments.

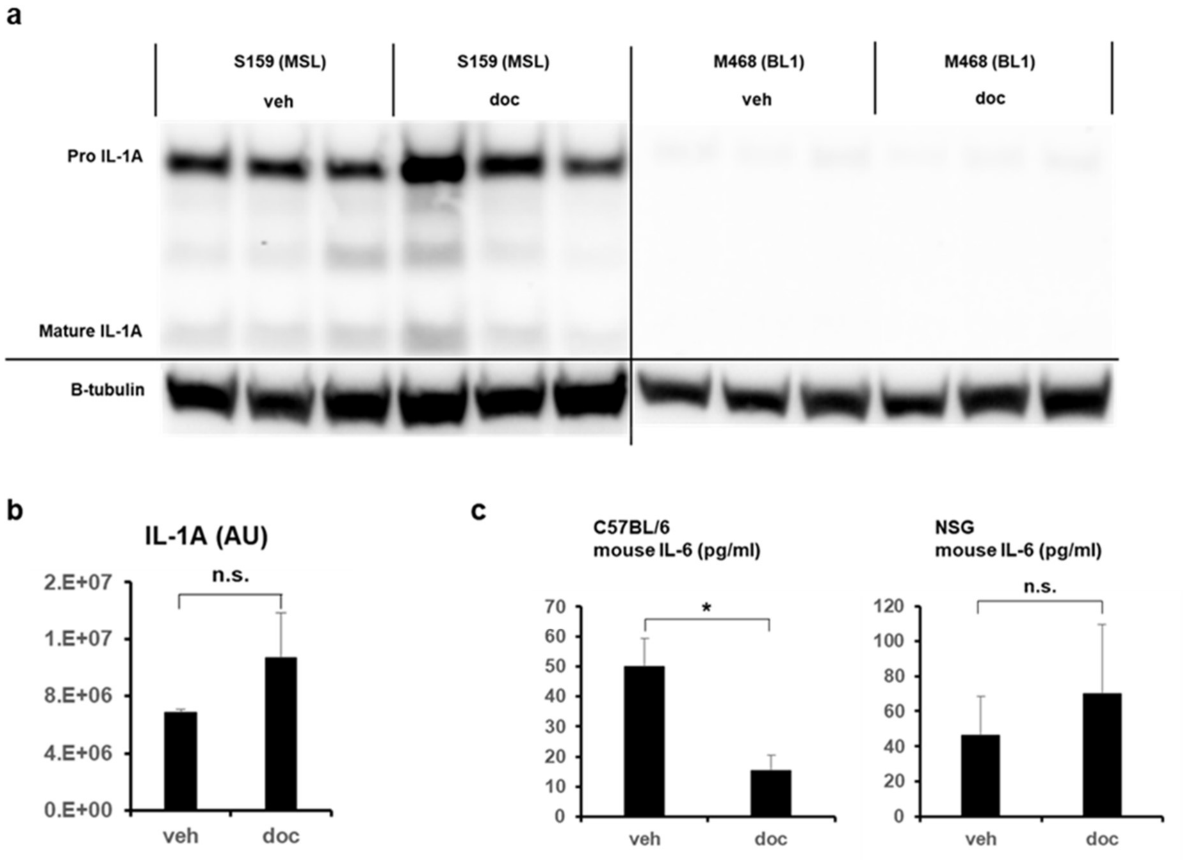

Supplementary Figure 7

Supplementary Figure 7. Docetaxel mediated induction of IL-6 is specific for human MSL TNBCs and not host murine IL-6 production. A. Female NSG mice were implanted with indicated human TNBC cell lines. On day zero, mice were treated with vehicle (veh) or docetaxel (doc) 20 mg/kg. On day two, mice were sacrificed and primary tumors were collected. Protein was isolated from tumor lysates and WB was performed. Shown are results from n=3 pairs of mice from both cell line xenografts. The six indicated MSL samples were performed on one membrane and the six BL1 samples were performed on a separate membrane. B. S159 cell line xenograft IL-1A densitometry average ± SEM comparing 3 pairs of animals. C. Female mice of indicated strains were treated with veh or doc (20 mg/kg). Two days later, serum was collected and circulating mouse IL-6 average ± SEM was measured by BD Bio mouse IL-6 ELISA kit. Both strains included four pairs of animals. Statistical analysis performed with unpaired t-test: $* p < 0.05$; $** p < 0.01$.

Supplementary Table 1.

primer	Sequence 5' -> 3'
Human IL-1A Forward	CGCCAATGACTCAGAGGAAGA
Human IL-1A Reverse	AGGGCGTCATTCAGGATGAA
Human IL-6 Forward	CAAATTCGGTACATCCTCGACGGC
Human IL-6 Reverse	GGTTCAGGTTGTTTTCTGCCAGTGC
Human COX-2 Forward	TGCATTCTTTGCCCAGCACT
Human COX-2 Reverse	AAAGGCGCAGTTTACGCTGT
Human TBP Forward	TTGGGTTTTCCAGCTAAGTTCT
Human TBP Reverse	CCAGGAAATAACTCTGGCTCA

Uncropped Western Blot Images - Main Figures:

Figure 1F

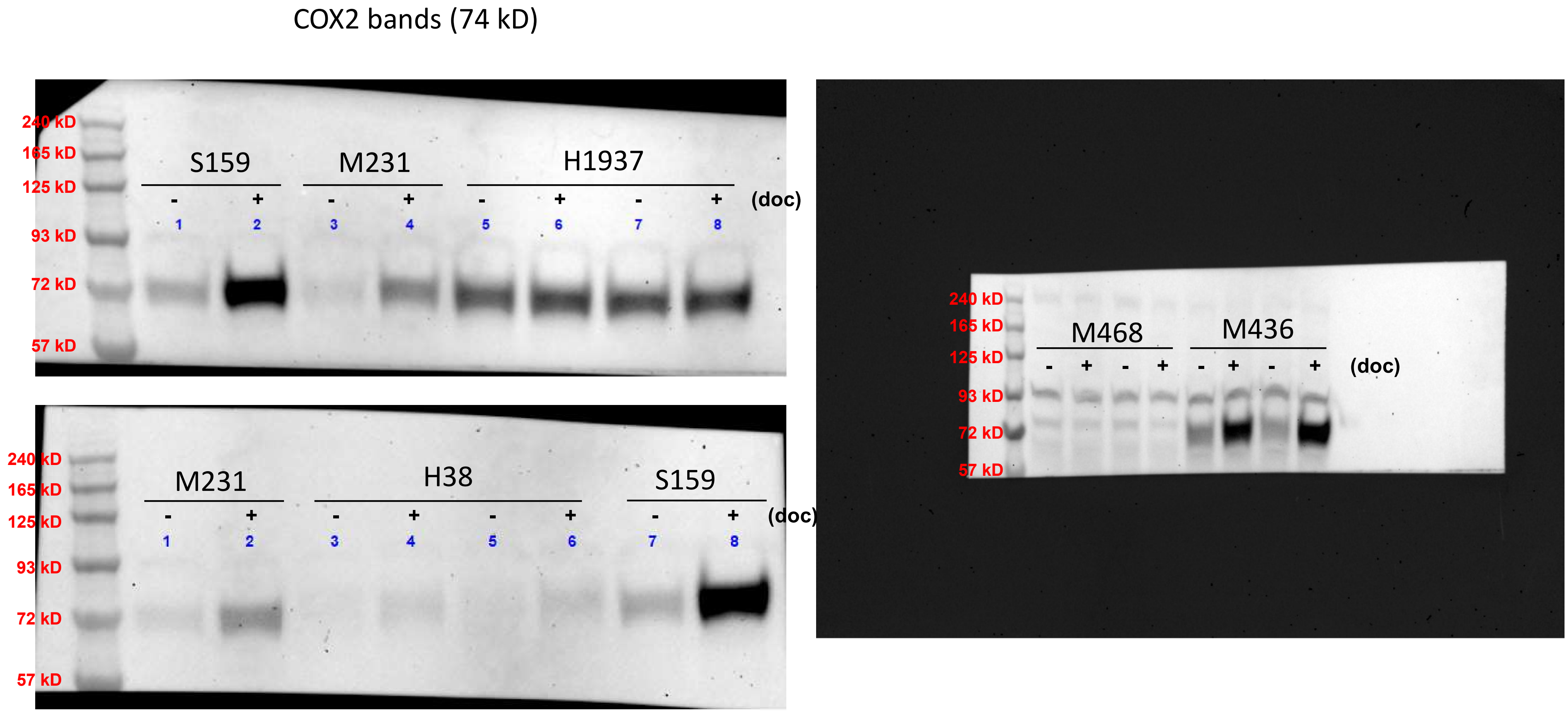

Figure 1F

B-actin bands 45 kD

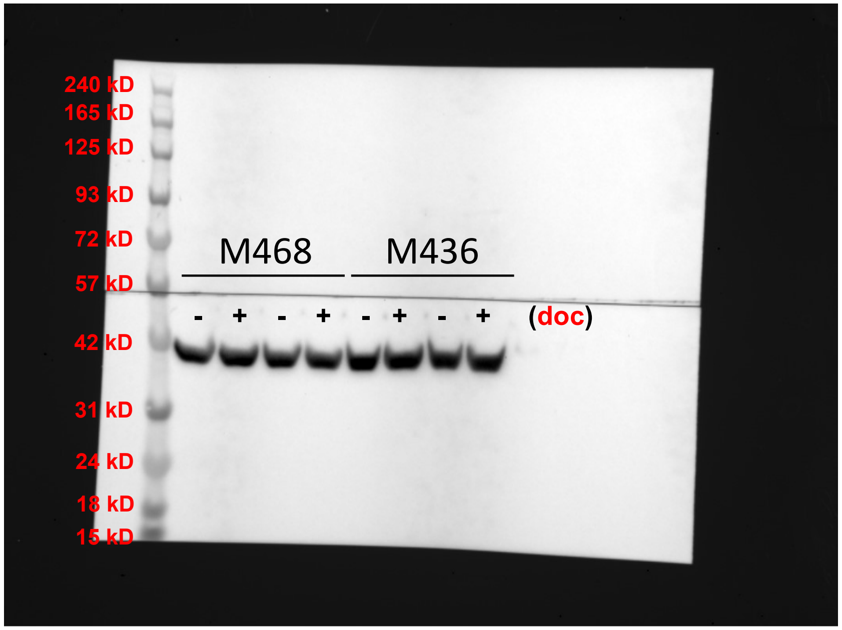

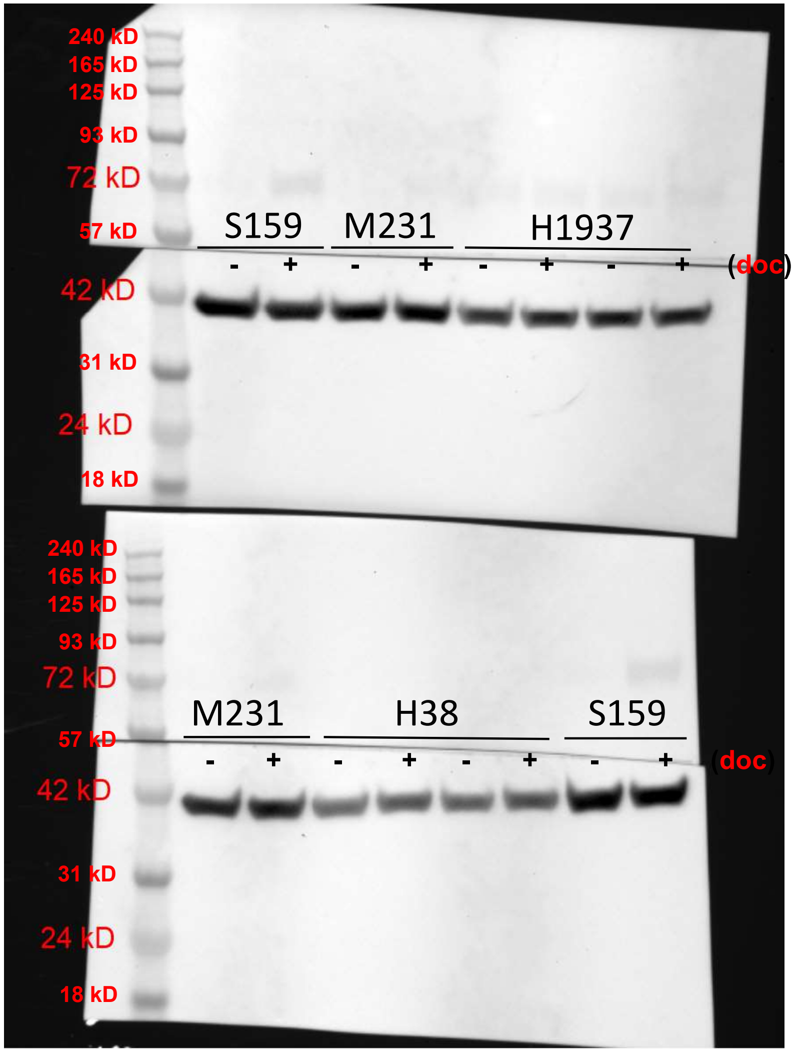

Figure 2C

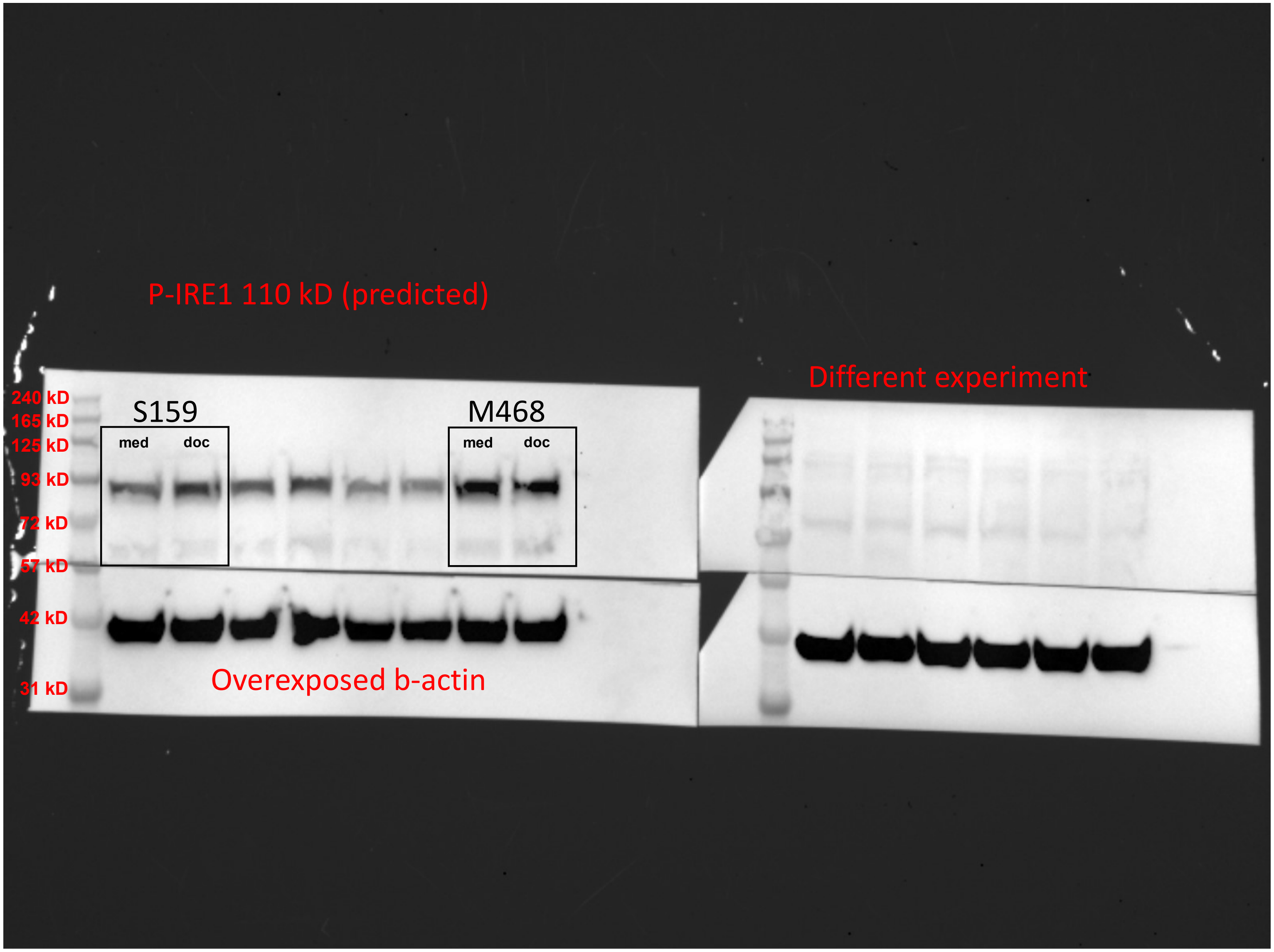
P-IRE1 110 kD (predicted)
240 kD
165 kD
125 kD
93 kD
72 kD
57 kD
42 kD
31 kD
S159
med
doc
M468
med
doc
Different experiment
Overexposed b-actin

Figure 2C

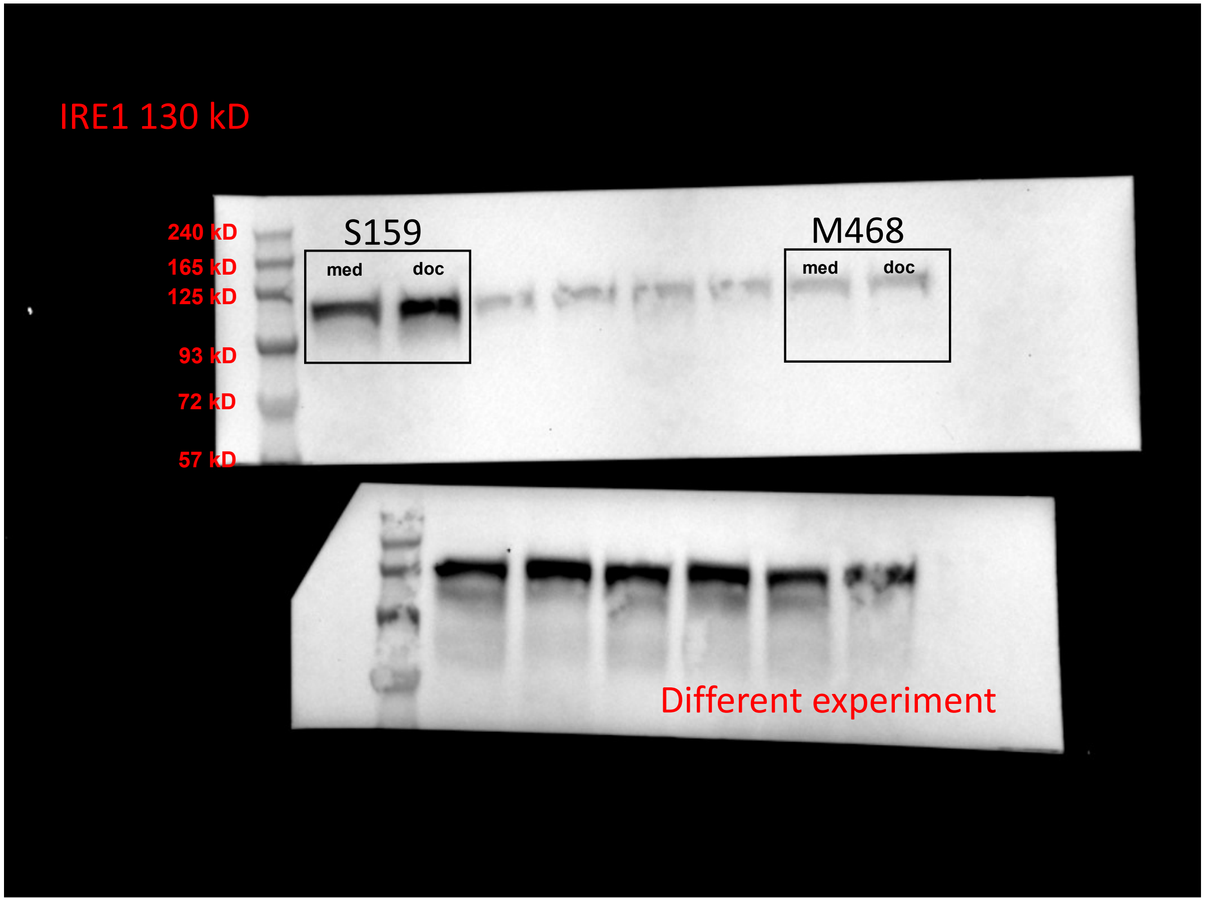

IRE1 130 kD
240 kD
165 kD
125 kD
93 kD
72 kD
57 kD
S159
med
doc
M468
med
doc
Different experiment

Figure 2C

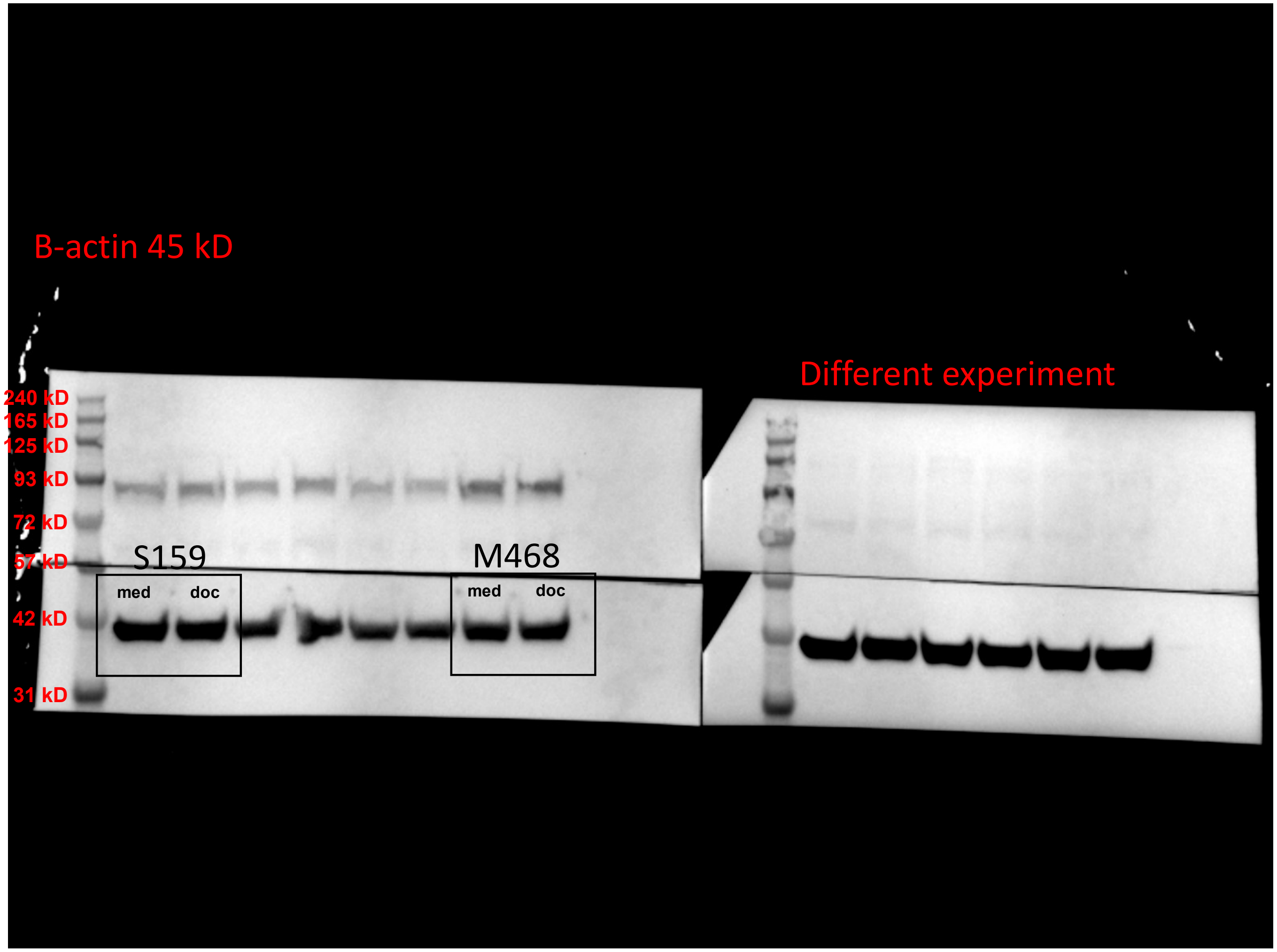

B-actin 45 kD
Different experiment
240 kD
165 kD
125 kD
93 kD
72 kD
57 kD
42 kD
31 kD
S159
M468
med
doc
med
doc

Figure 3A

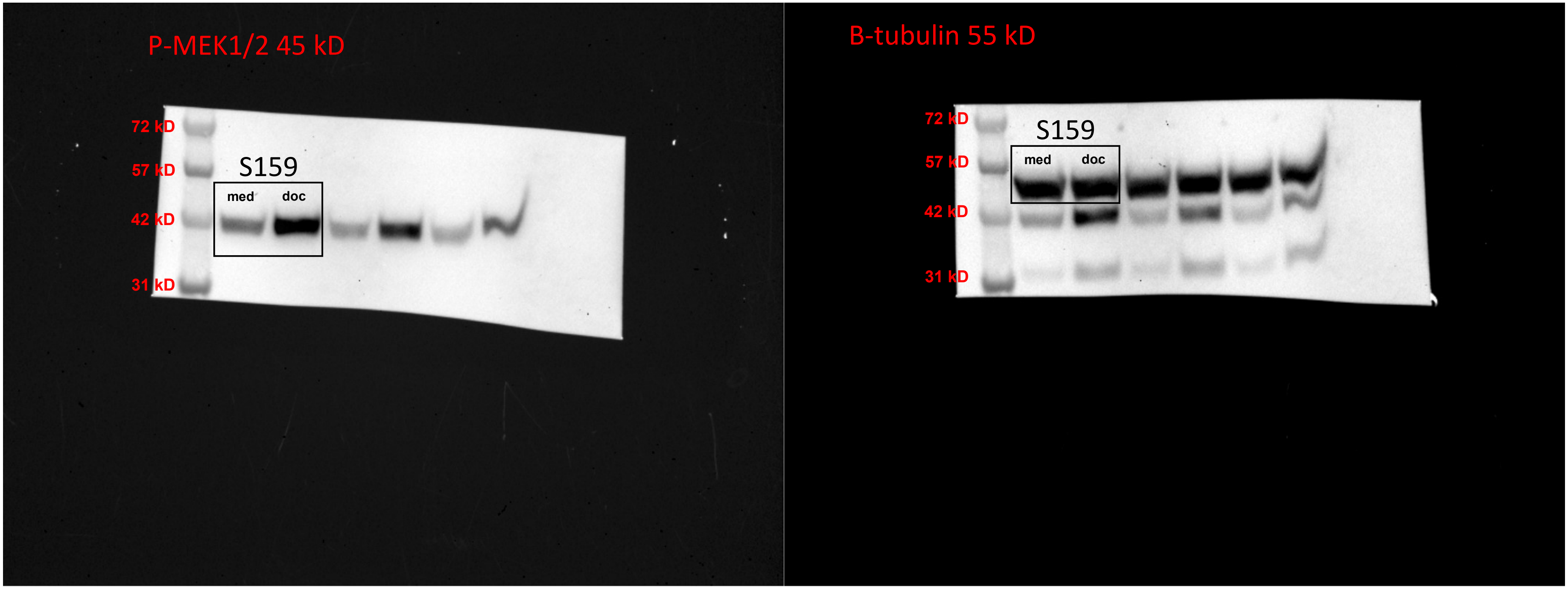

P-MEK1/2 45 kD
72 kD
57 kD
42 kD
31 kD
S159
med
doc
B-tubulin 55 kD
72 kD
57 kD
42 kD
31 kD
S159
med
doc

Figure 3A

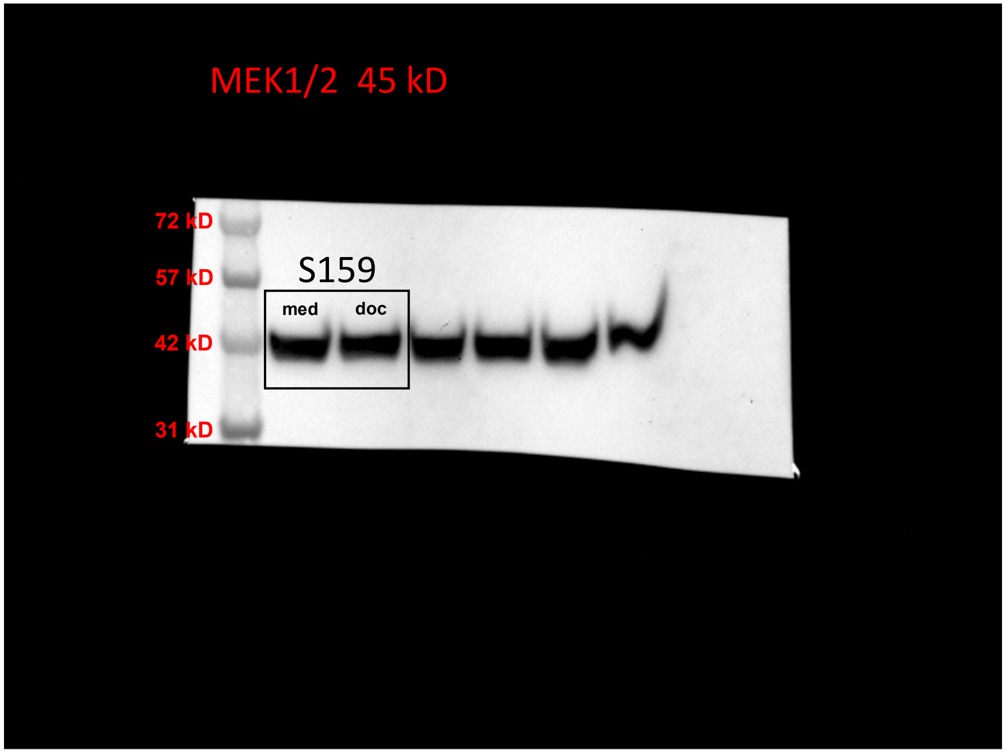

MEK1/2 45 kD
72 kD
57 kD
42 kD
31 kD
S159
med
doc

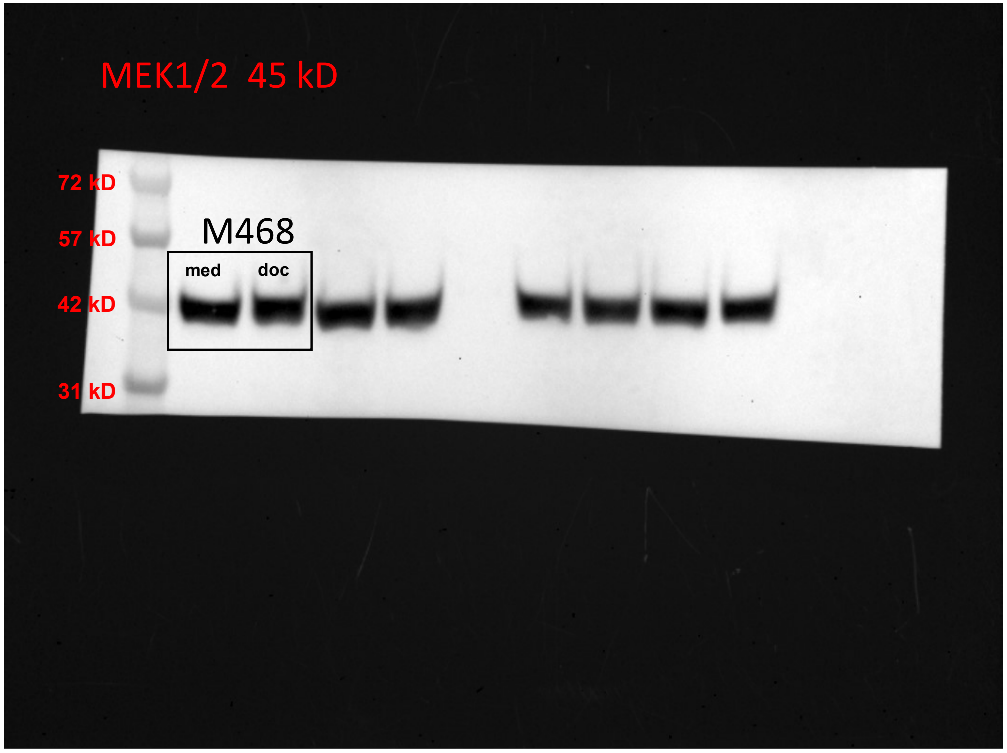

MEK1/2 45 kD
72 kD
57 kD
42 kD
31 kD
M468
med
doc

Figure 3A

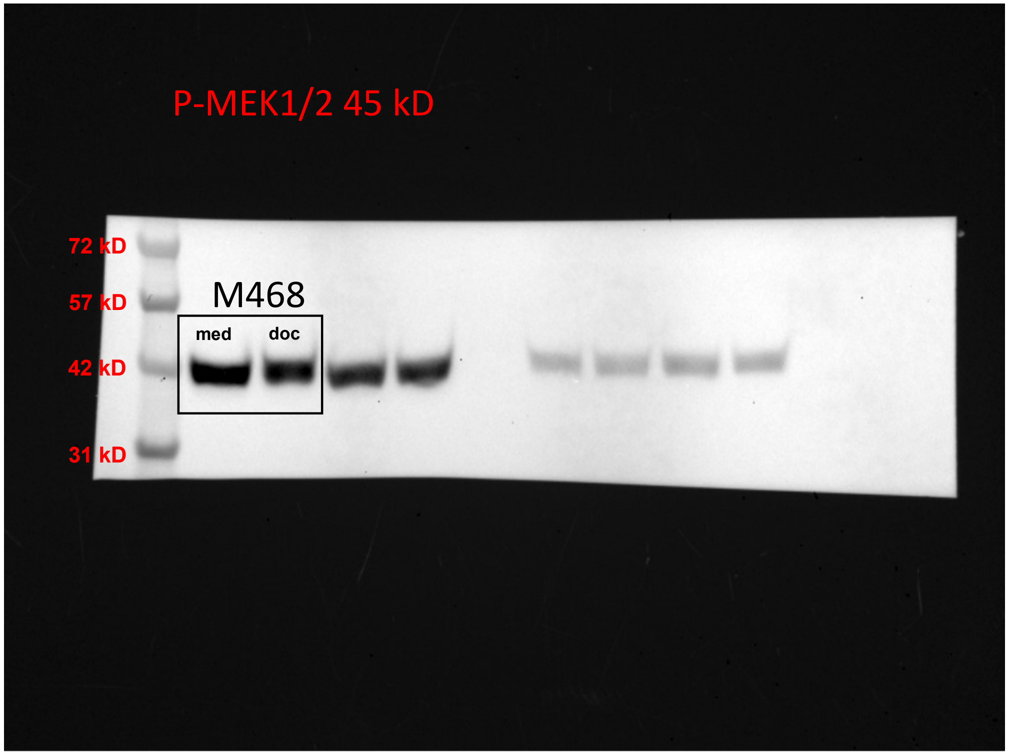

P-MEK1/2 45 kD
72 kD
57 kD
42 kD
31 kD
M468
med
doc

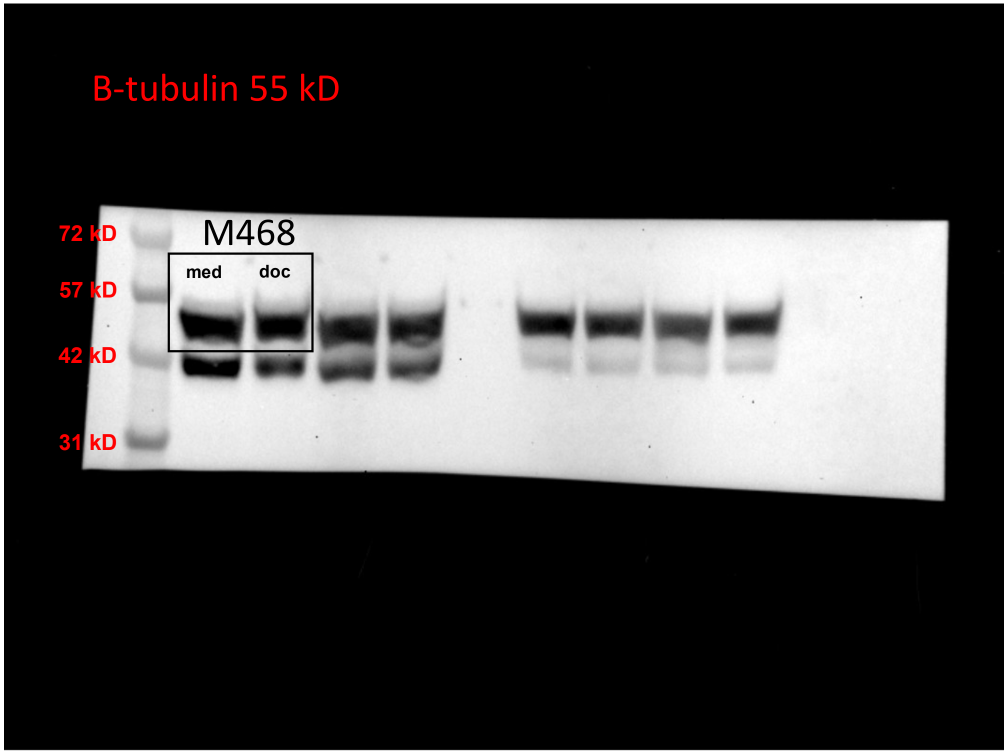

B-tubulin 55 kD
72 kD
57 kD
42 kD
31 kD
M468
med
doc

Figure 3D

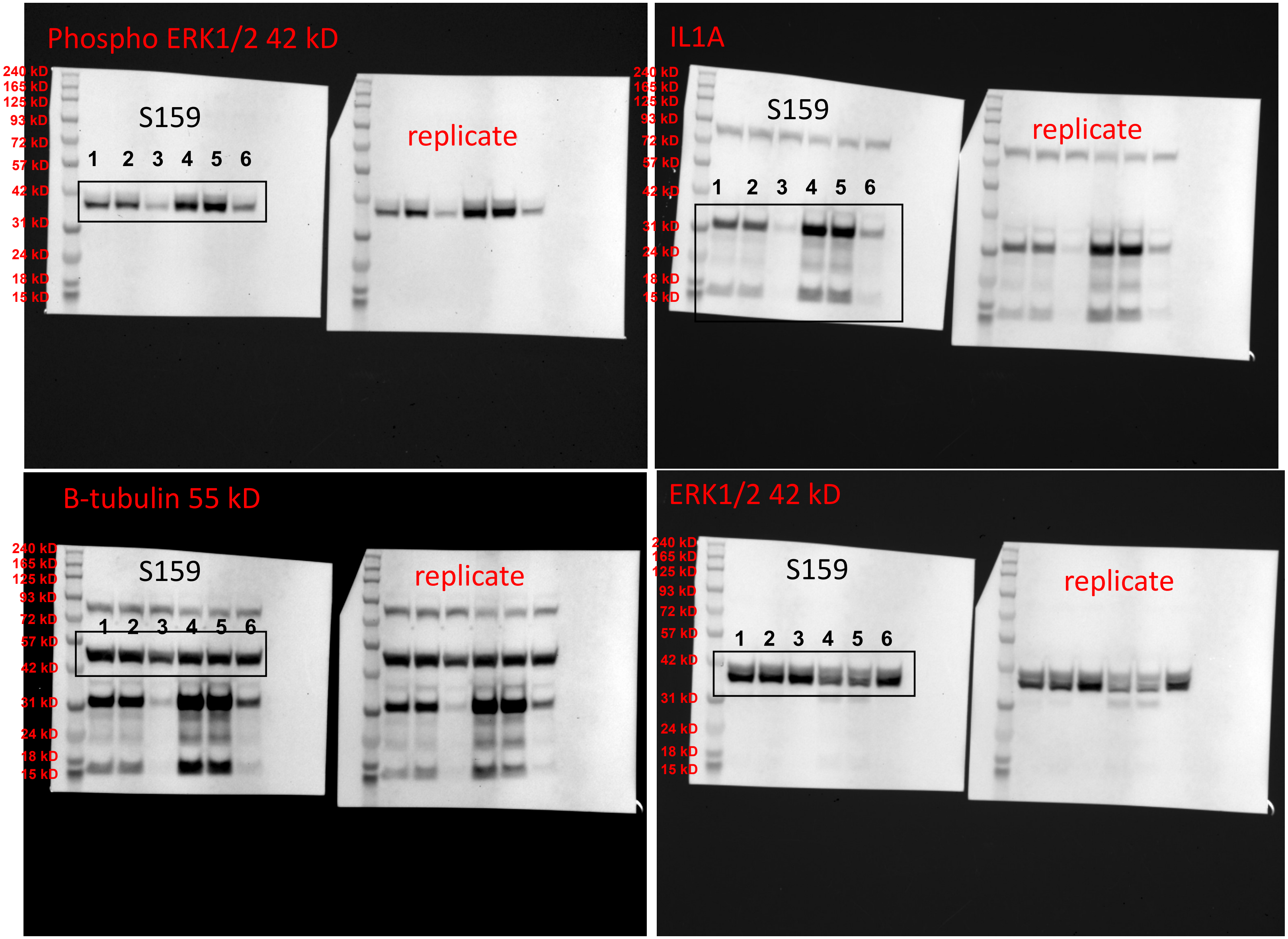

1. Media
2. DMSO
3. PD03
4. Doc
5. Doc + DMSO
6. Doc + PD03

Figure 4A

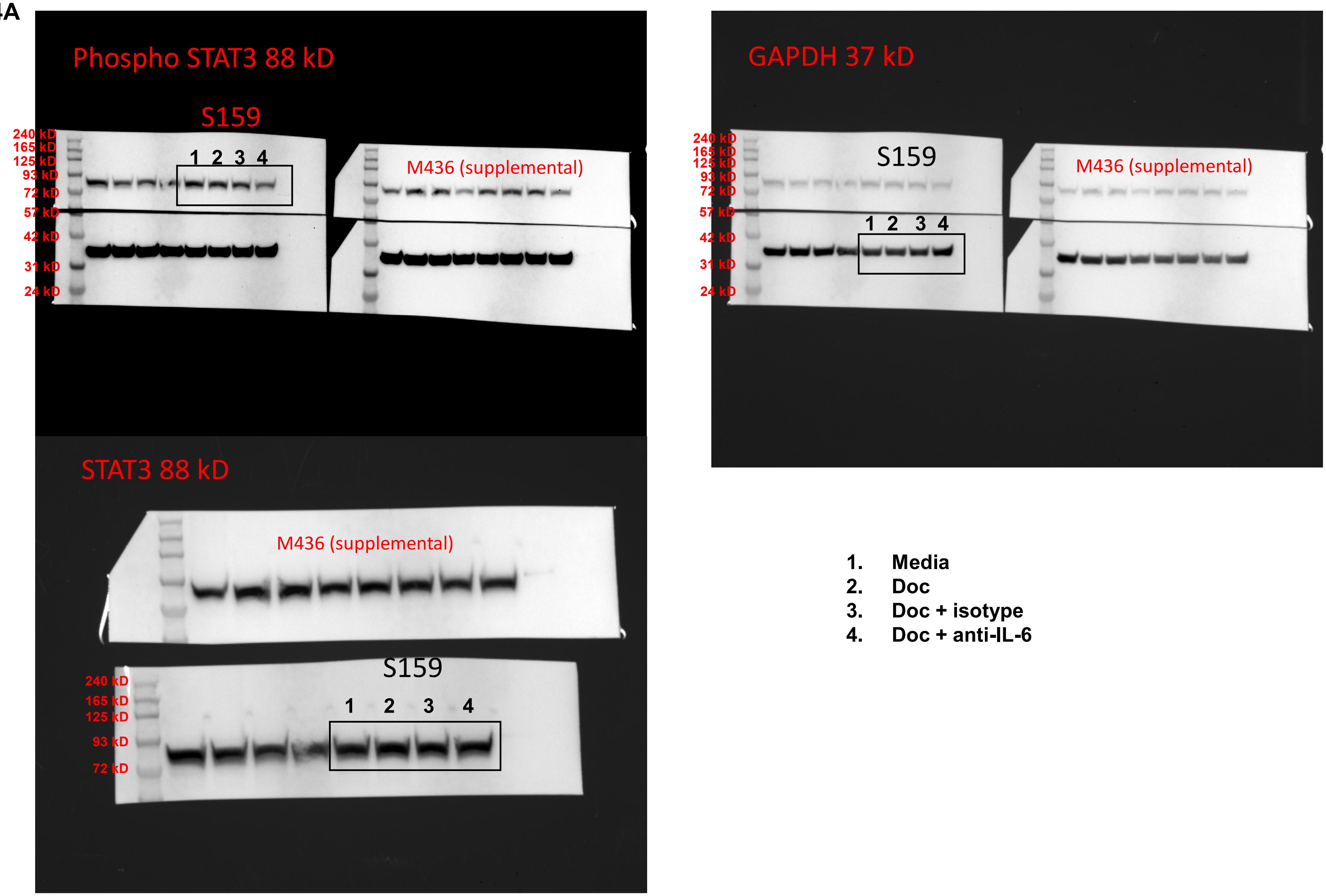

1. **Media**
2. **Doc**
3. **Doc + isotype**
4. **Doc + anti-IL-6**

Figure 4A

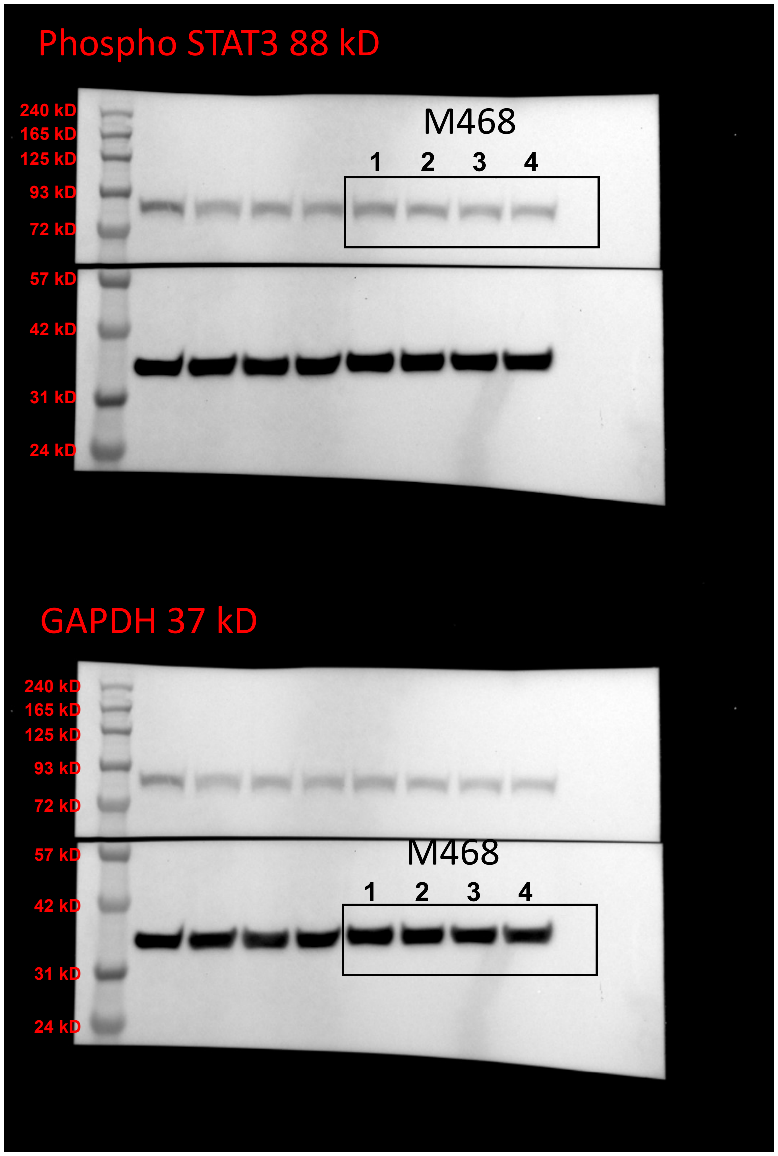

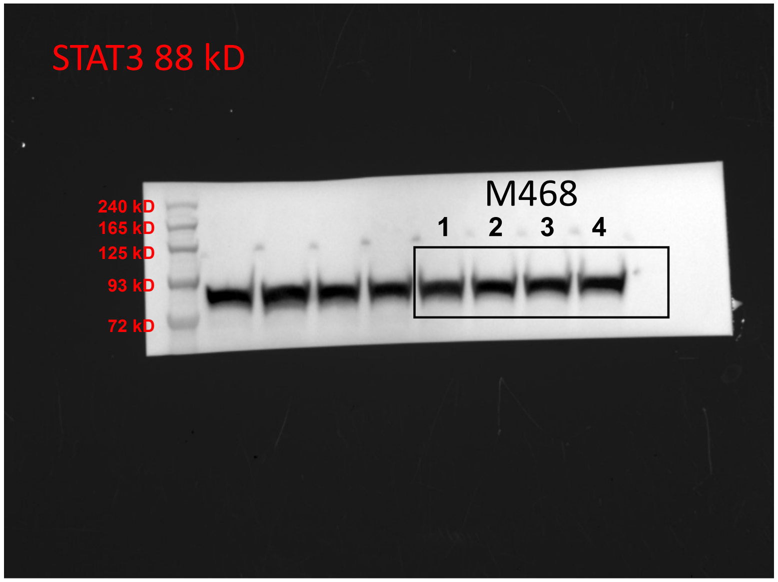

1. Media
2. Doc
3. Doc + isotype
4. Doc + anti-IL-6

Figure 5A

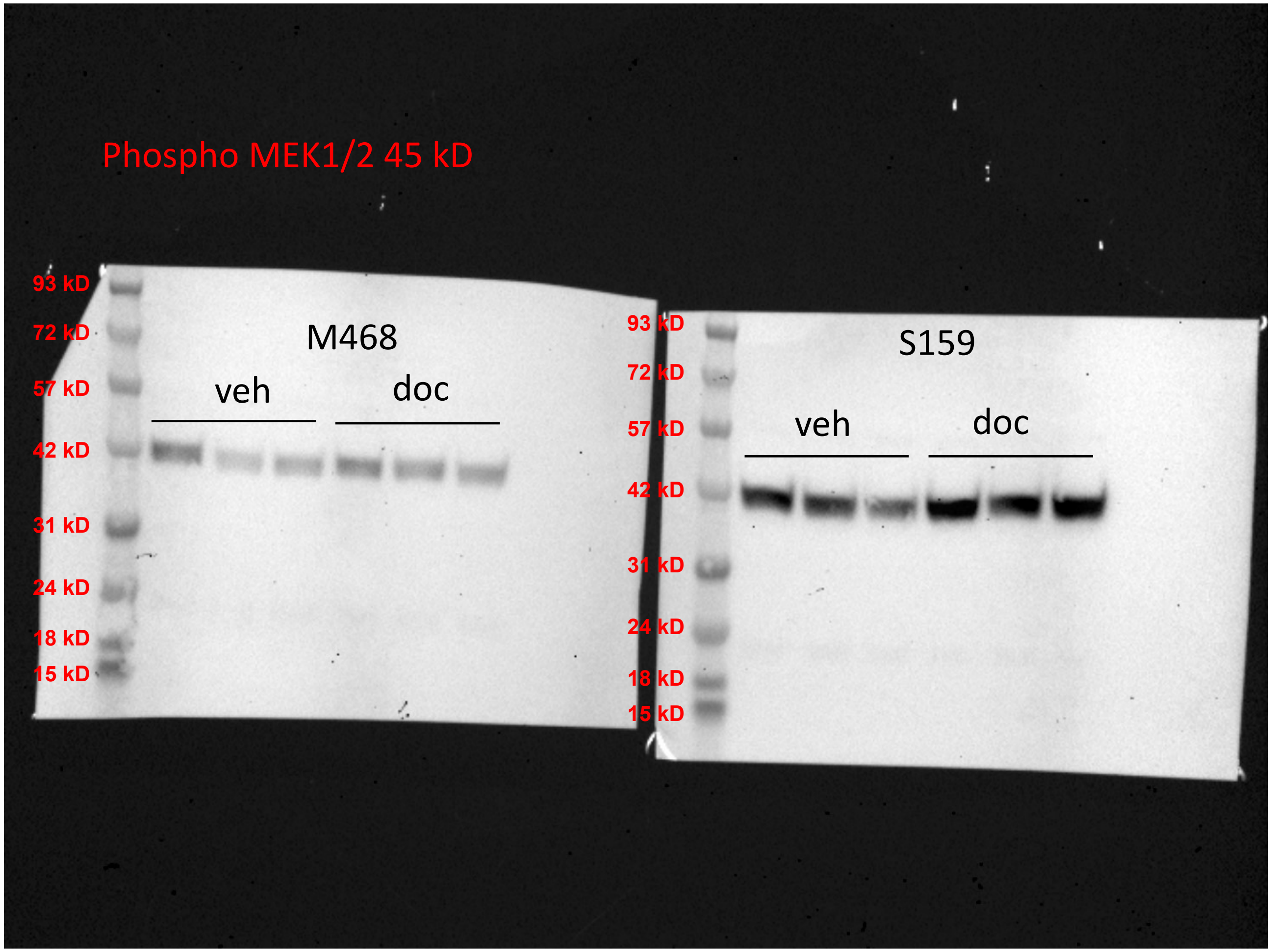
Phospho MEK1/2 45 kD
M468
veh
doc
93 kD
72 kD
57 kD
42 kD
31 kD
24 kD
18 kD
15 kD
S159
veh
doc
93 kD
72 kD
57 kD
42 kD
31 kD
24 kD
18 kD
15 kD

Figure 5A

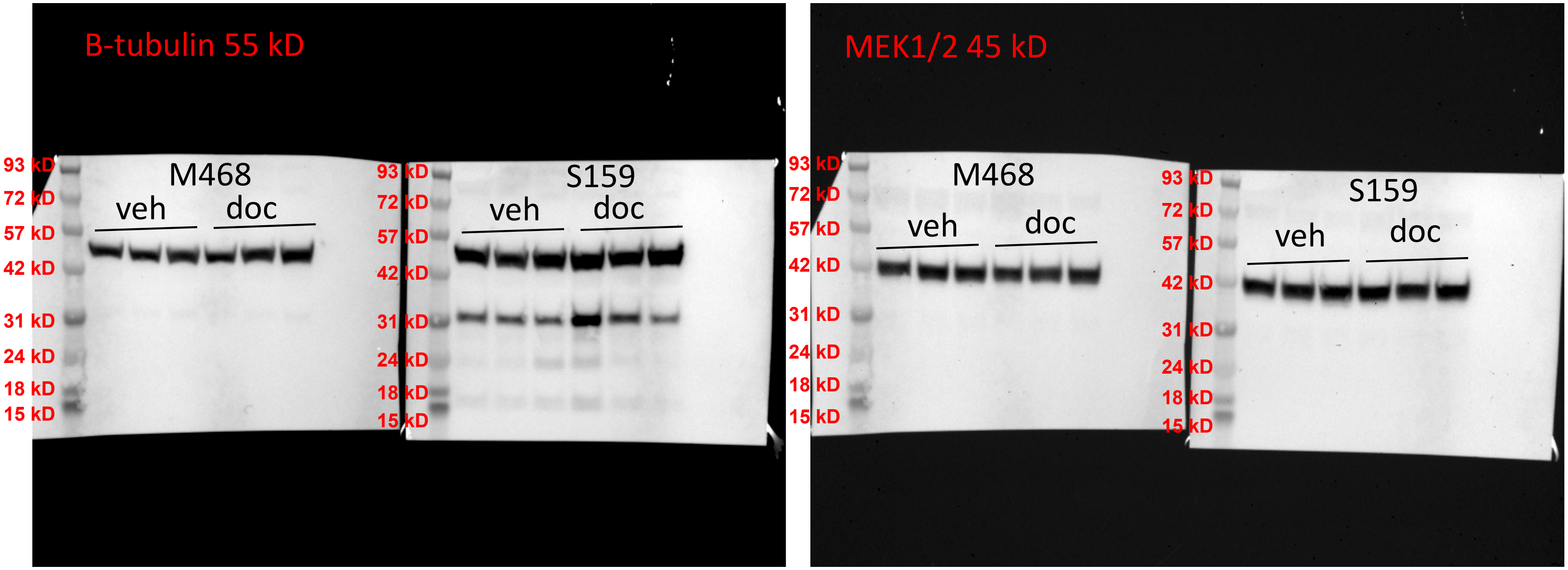

B-tubulin 55 kD
M468
veh
doc
S159
veh
doc
93 kD
72 kD
57 kD
42 kD
31 kD
24 kD
18 kD
15 kD
MEK1/2 45 kD
M468
veh
doc
S159
veh
doc

Uncropped Western Blot Images - Supplementary Figures:

Supplementary Figure 2

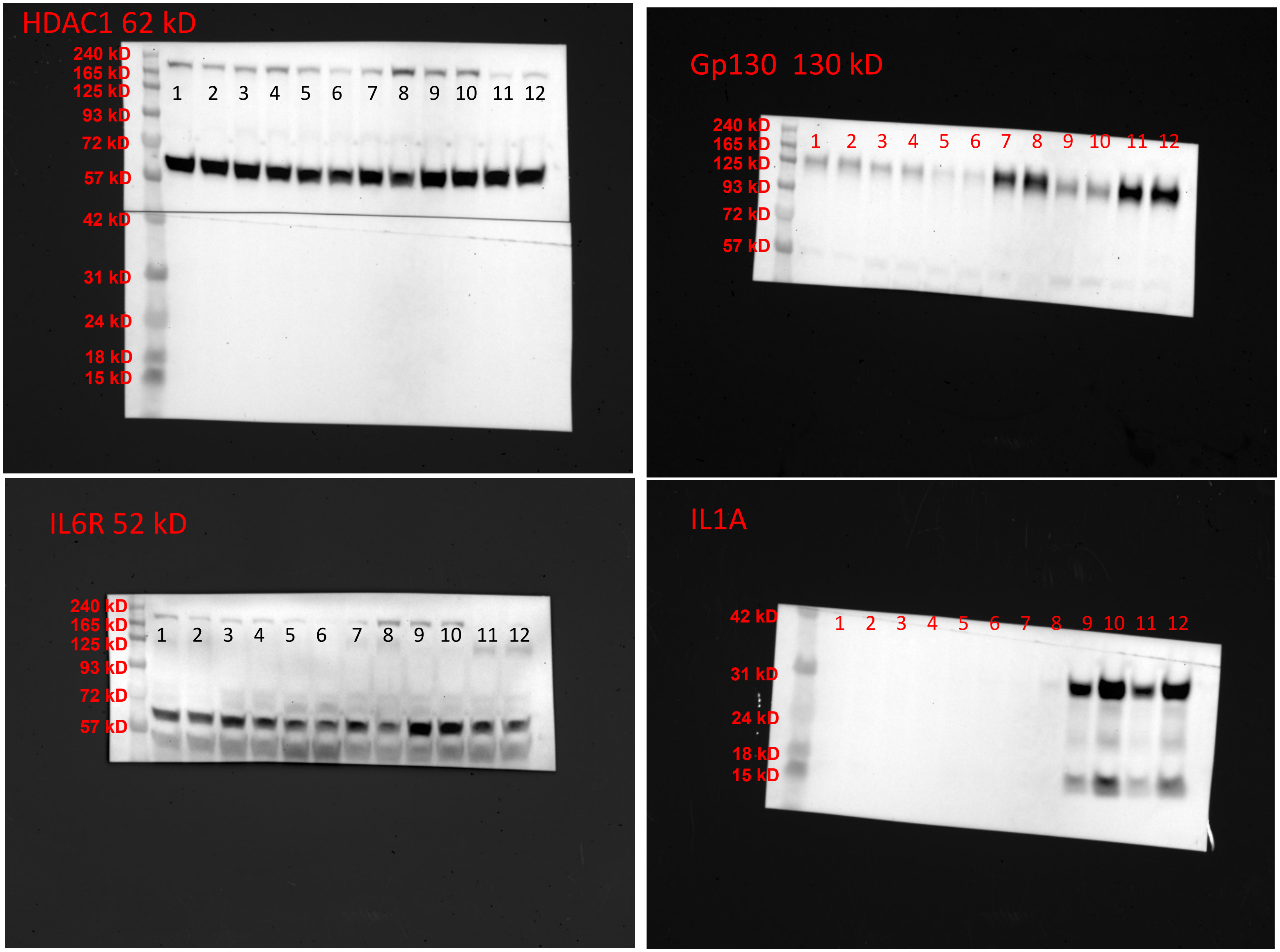

1. H38 med
2. H38 doc
3. M468 med
4. M468 doc
5. H1937 med
6. H1937 doc
7. M231 med
8. M231 doc
9. M436 med
10. M436 doc
11. S159 med
12. S159 doc

Supplementary Figure 3

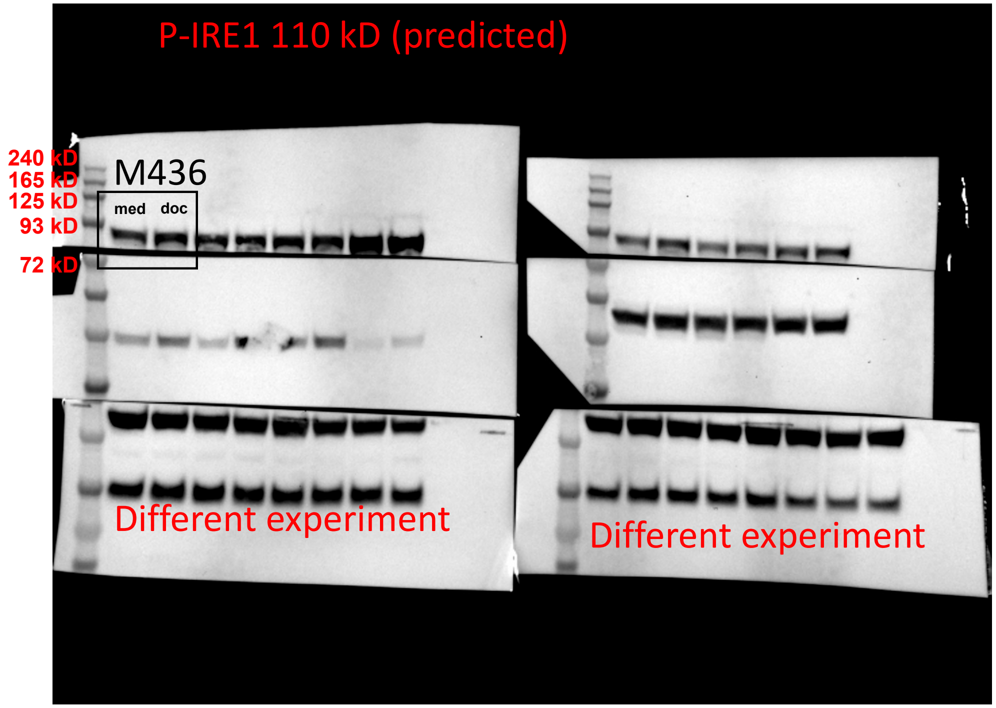

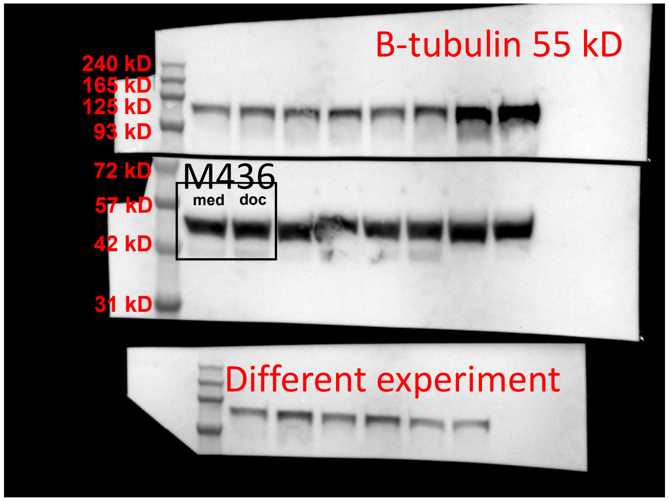

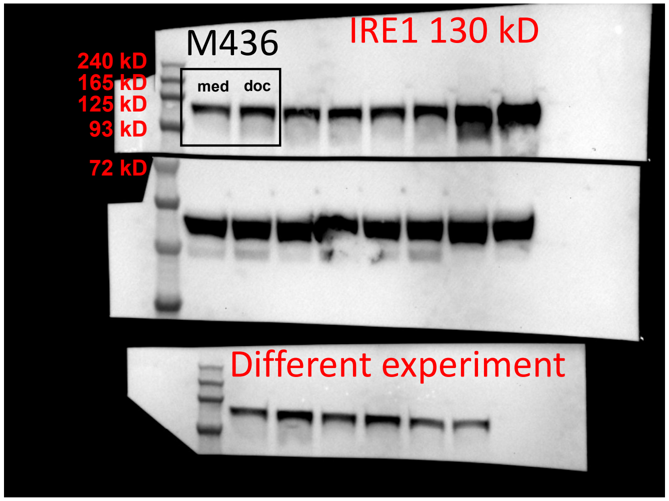

Supplementary Figure 3

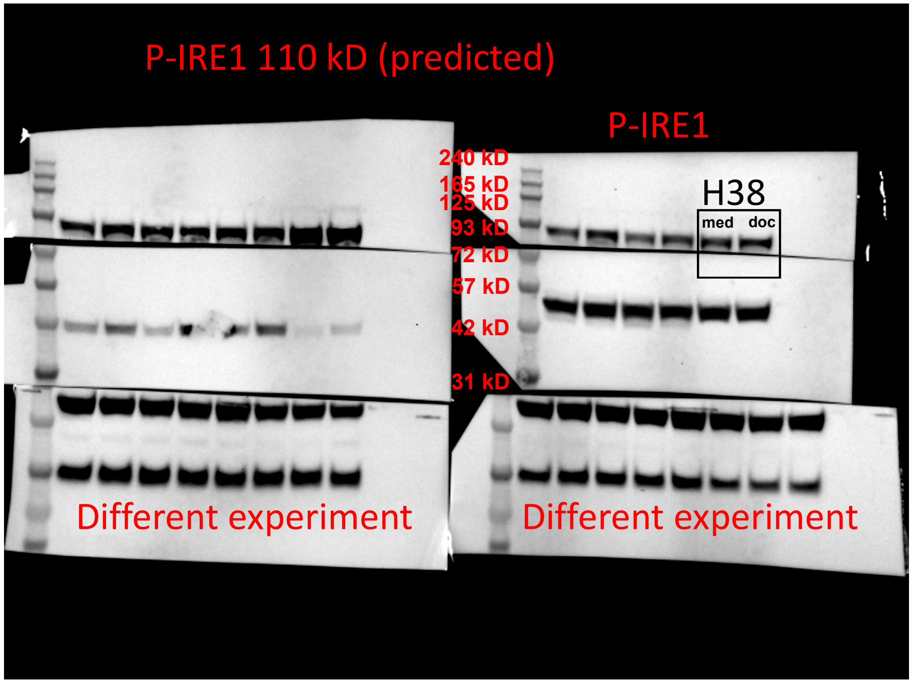

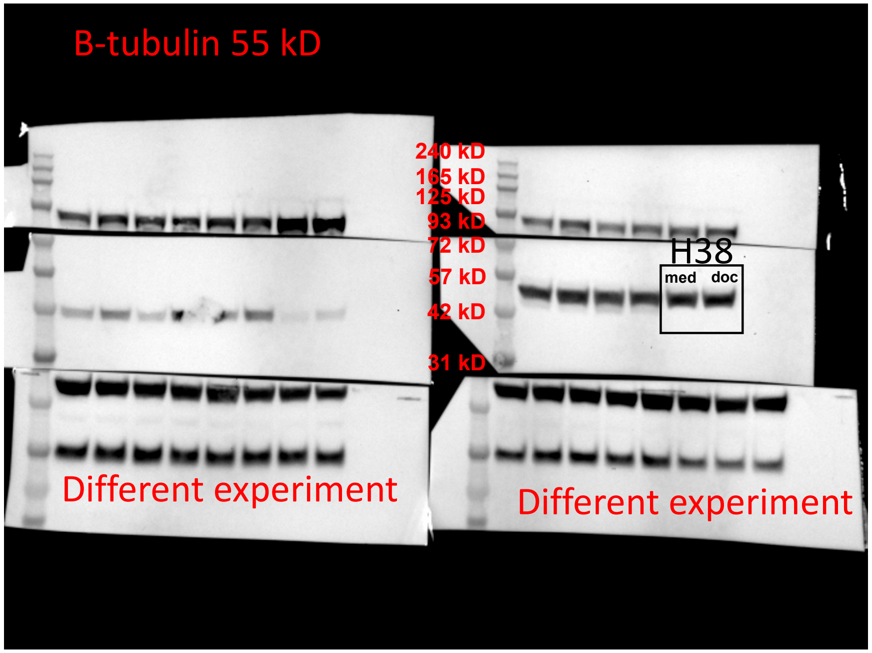

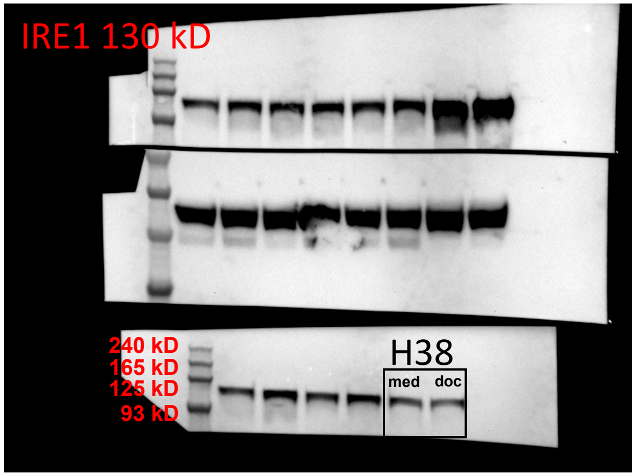

Supplementary Figure 4

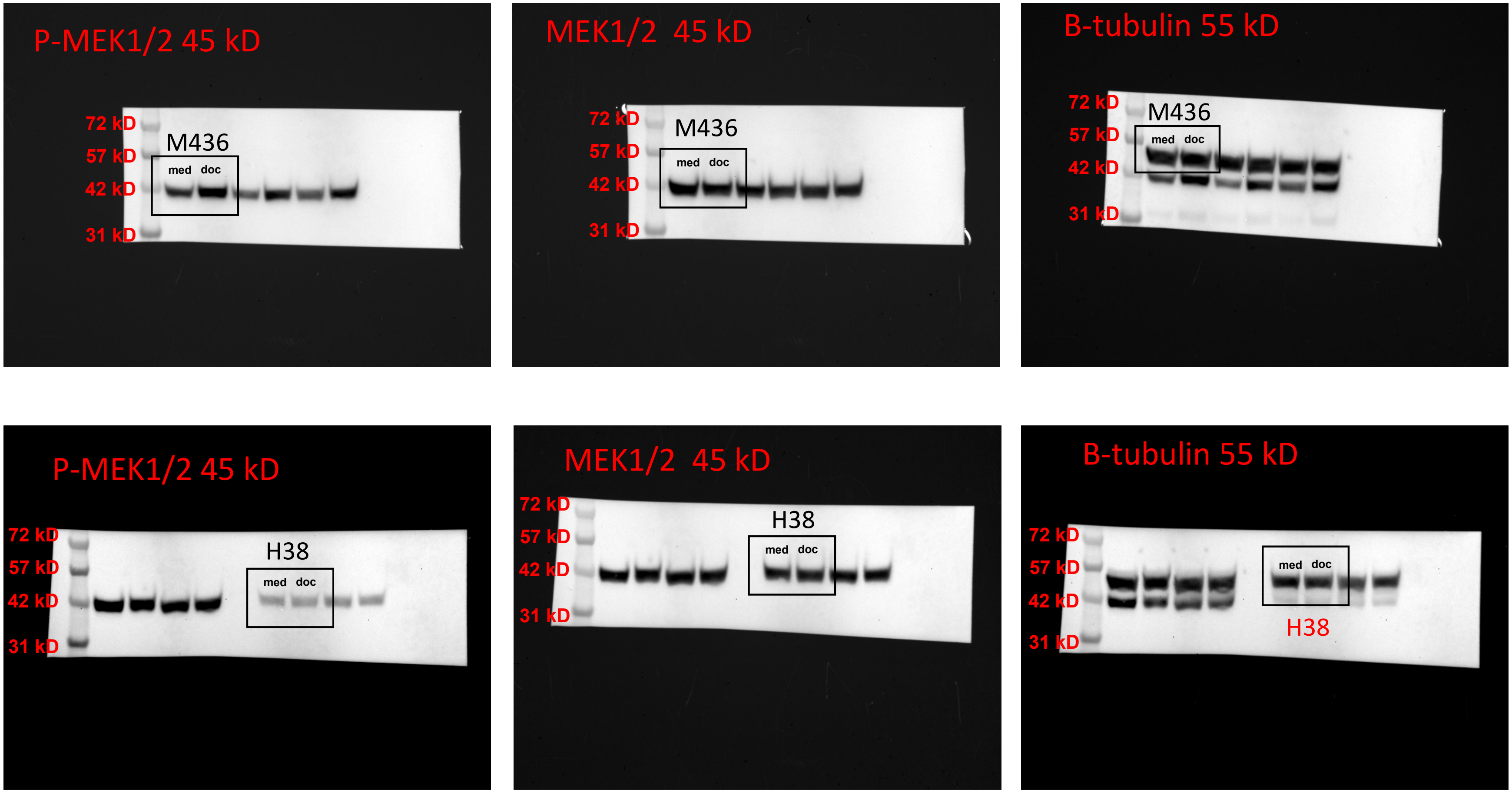

Supplementary Figure 4

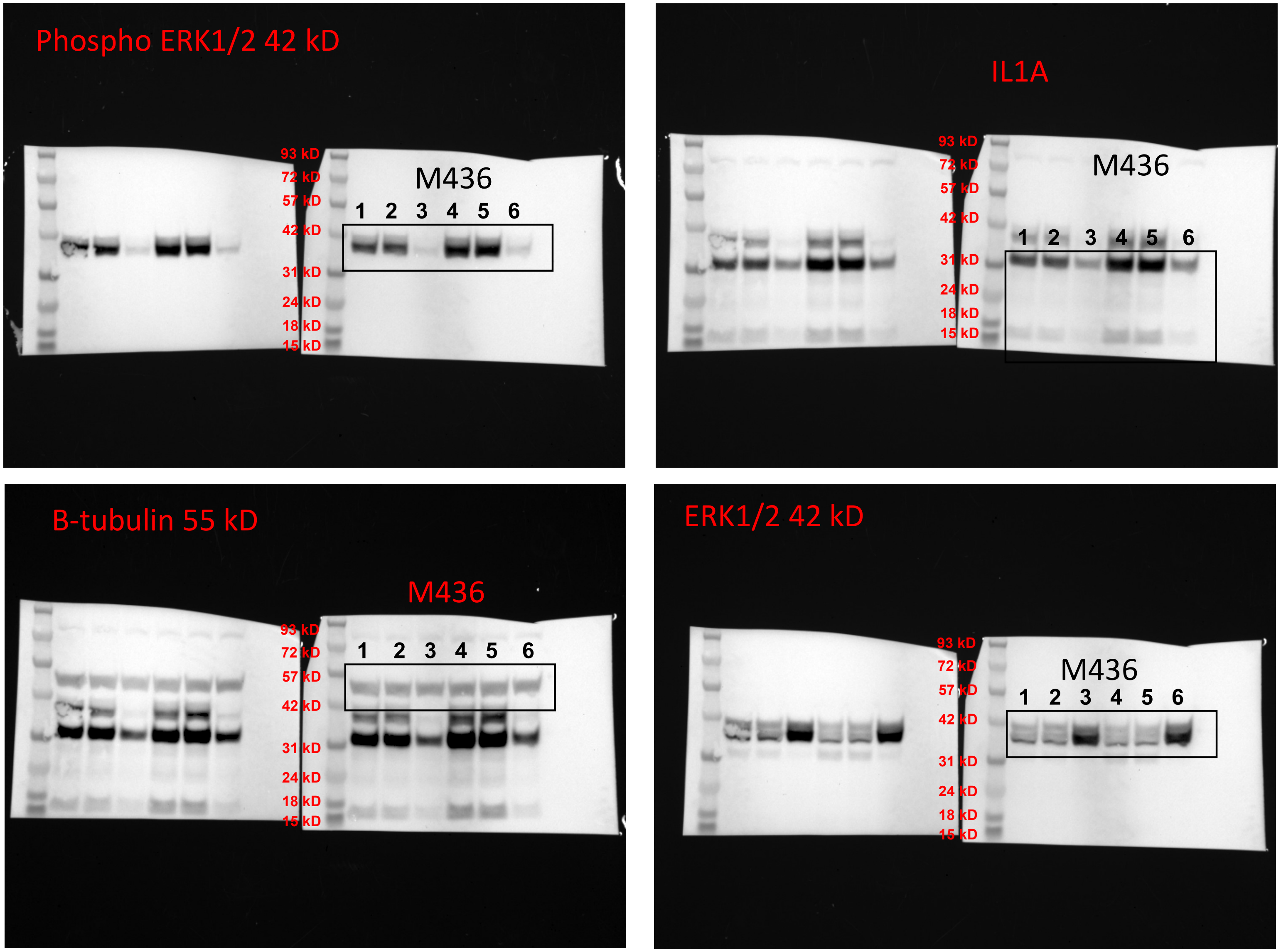

1. Media
2. DMSO
3. PD03
4. Doc
5. Doc + DMSO
6. Doc + PD03

Supplementary Figure 6

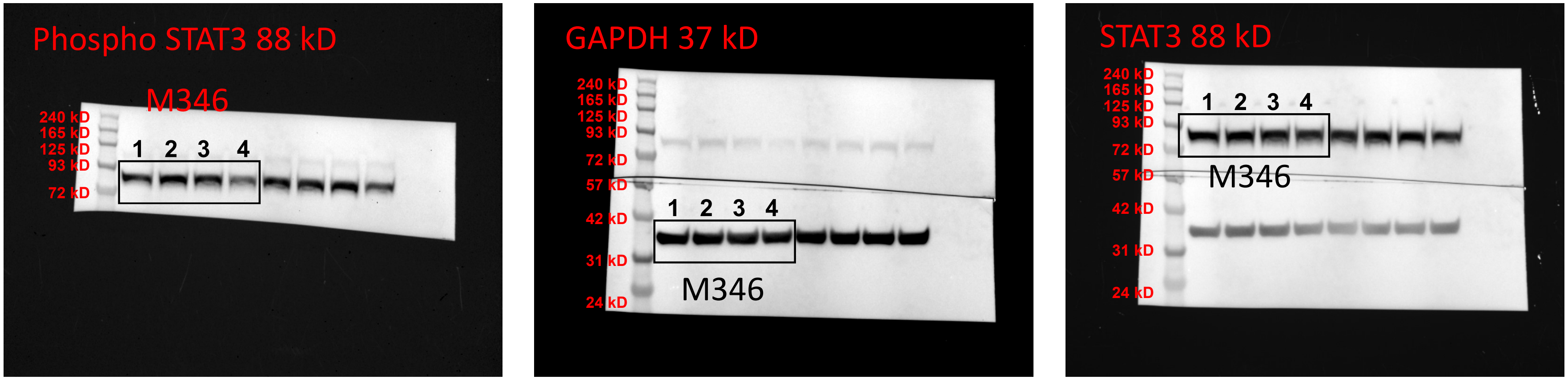

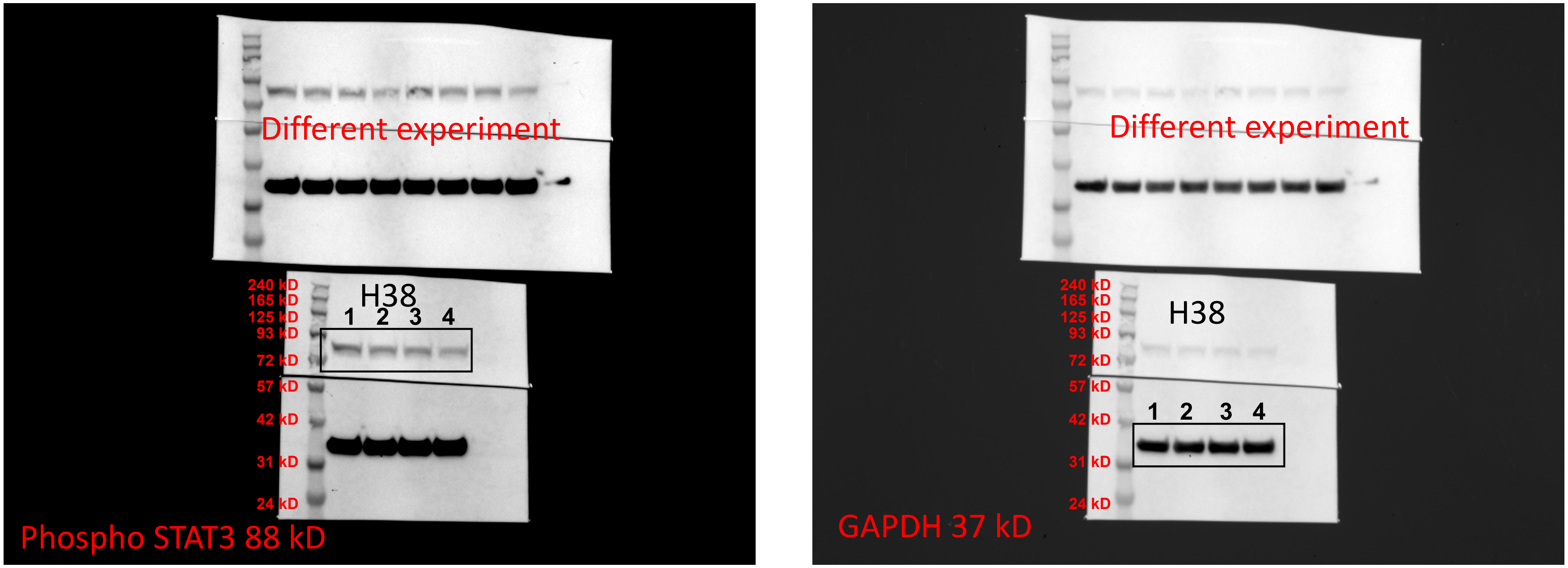

1. Media
2. Doc
3. Doc + isotype
4. Doc + anti-IL-6

Supplementary Figure 6

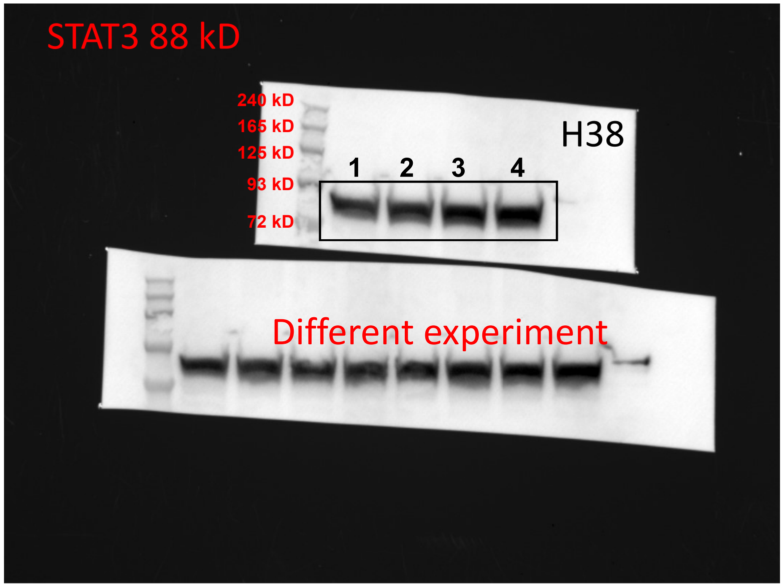

1. **Media**
2. **Doc**
3. **Doc + isotype**
4. **Doc + anti-IL-6**

Supplementary Figure 7

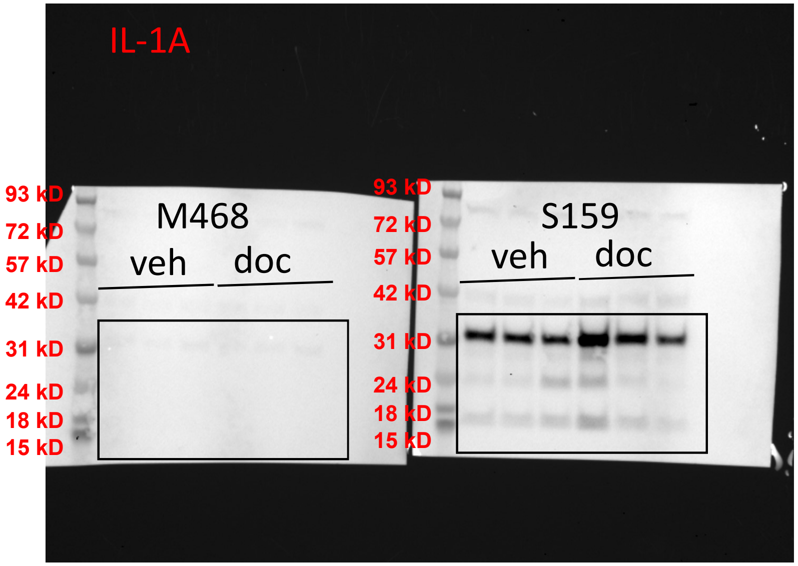

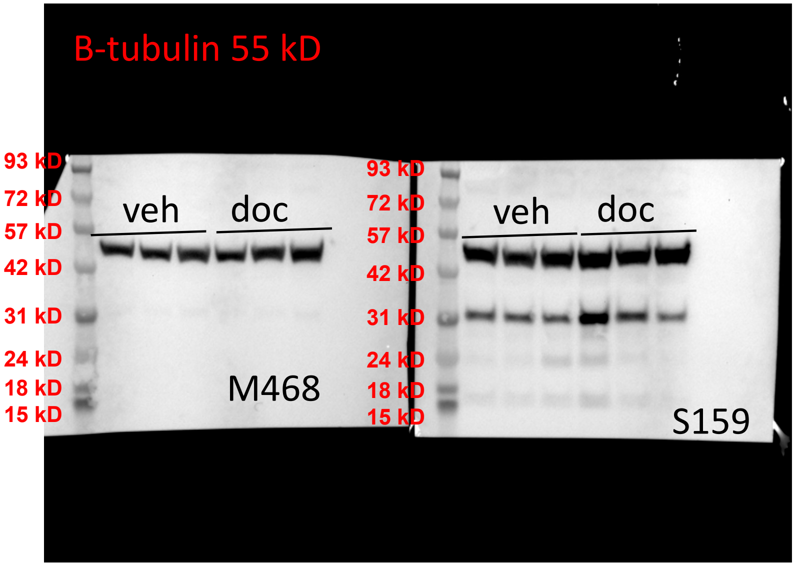

Supplement: Supplementary file 1 — Supplmental material [file 41523_2021_371_MOESM1_ESM.pdf]
